# Development of an *in vitro* method for activation of X-succinate synthases for fumarate hydroalkylation

**DOI:** 10.1016/j.isci.2023.106902

**Published:** 2023-05-19

**Authors:** Mary C. Andorfer, Devin T. King-Roberts, Christa N. Imrich, Balyn G. Brotheridge, Catherine L. Drennan

**Affiliations:** 1 Department of Biology, Massachusetts Institute of Technology, Cambridge, MA 02139, USA; 2 Department of Biological Engineering, Massachusetts Institute of Technology, Cambridge, MA 02139, USA; 3 Howard Hughes Medical Institute, Massachusetts Institute of Technology, Cambridge, MA 02139, USA; 4 Department of Chemistry, Massachusetts Institute of Technology, Cambridge, MA 02139, USA; 5 Center for Environmental Health, Massachusetts Institute of Technology, Cambridge, MA 02139, USA; 6 Bio-inspired Solar Energy Program, Canadian Institute for Advanced Research (CIFAR), Toronto, ON M5G 1M1, Canada

**Keywords:** Biochemistry, Biocatalysis, Structural biology, Bioengineering

## Abstract

Anaerobic microbial degradation of hydrocarbons is often initiated through addition of the hydrocarbon to fumarate by enzymes known as X-succinate synthases (XSSs). XSSs use a glycyl radical cofactor, which is installed by an activating enzyme (XSS-AE), to catalyze this carbon-carbon coupling reaction. The activation step, although crucial for catalysis, has not previously been possible *in vitro* because of insolubility of XSS-AEs. Here, we take a genome mining approach to find an XSS-AE, a 4-isopropylbenzylsuccinate synthase (IBSS)-AE (IbsAE) that can be solubly expressed in *Escherichia coli*. This soluble XSS-AE can activate both IBSS and the well-studied benzylsuccinate synthase (BSS) *in vitro*, allowing us to explore XSSs biochemically. To start, we examine the role of BSS subunits and find that the beta subunit accelerates the rate of hydrocarbon addition. Looking forward, the methodology and insight gathered here can be used more broadly to understand and engineer XSSs as synthetically useful biocatalysts.

## Highlights

• X-succinate synthases (XSSs) use a glycyl radical to functionalize hydrocarbons
• Soluble XSS activating enzymes (XSS-AEs) were found through genome mining
• A soluble XSS-AE allowed *in vitro* glycyl radical installation within XSSs
• An auxiliary subunit is critical for fumarate hydroalkylation using XSSs

## Graphical abstract

**Figure:**
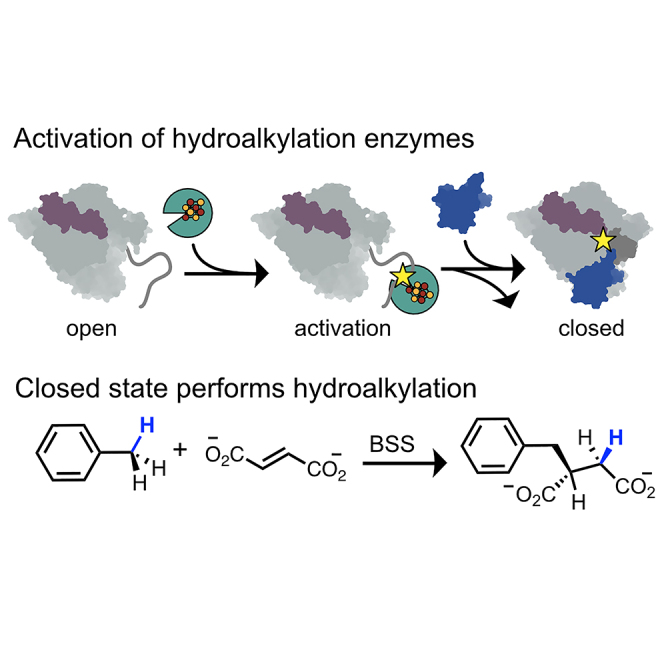

## Introduction

Hydrocarbons are abundant within both natural (e.g., marine hydrocarbon seeps) and artificial (e.g., oil pipelines) environments. Aerobic microbial degradation of hydrocarbons has been well characterized and used in bioremediation of crude-oil-polluted environments; however, hydrocarbons inevitably end up in marine and terrestrial anoxic environments as well.^1^^,^^2^ Even within oxic zones, intensive respiration of facultative microbes creates anoxic microenvironments. Further understanding of how hydrocarbons are anaerobically degraded by microbes is necessary to create better tools for bioremediation and to inhibit microbial corrosion^3^ within crude-oil-containing facilities. Characterization of microbial communities within these anaerobic environments remains an important, active area of research,^2^ and as more anaerobic degraders are discovered, it is also critical that we understand the underlying molecular mechanisms that allow these microbes to accomplish hydrocarbon degradation in the absence of molecular oxygen.^1^ One of the key reactions catalyzed by these organisms involves metabolism of hydrocarbon substrates via addition to fumarate. This hydroalkylation reaction proceeds via homolytic cleavage of a C–H bond on the hydrocarbon substrate, addition of the hydrocarbyl radical to the C=C bond of fumarate, and addition of hydrogen to the resulting succinyl radical. Beyond environmental significance, this type of reaction is a synthetically attractive method for forming C–C bonds, as it has the potential to rapidly build structural complexity within small molecules without byproducts or pre-functionalized substrates. The growing class of enzymes that catalyze this impressive reaction is known as the X-succinate synthases (XSSs).

**Figure 1:**
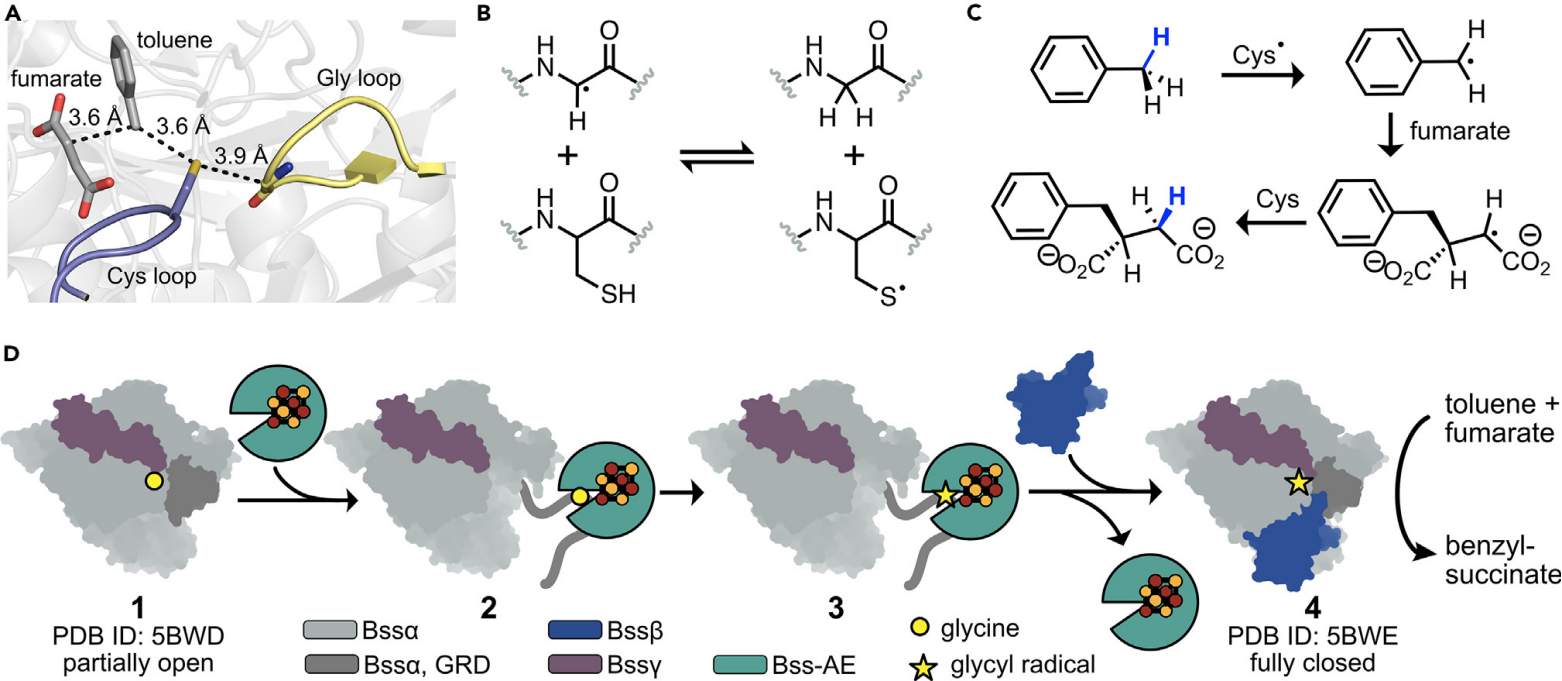
Overview of BSS mechanism X-ray crystallography of benzylsuccinate synthase (BSS) has provided insight into the molecular mechanisms involved in fumarate addition to toluene.^6^^,^^7^ (A) Snapshots of the active site of BSS (PDB: 5BWE)^7^ show how substrates are positioned for radical catalysis. The glycyl radical cofactor is harbored within the Gly loop (yellow), which is proximal to the Cys loop (purple). (B) The glycyl radical is proposed to be in equilibrium with a thiyl radical formed on the Cys residue within the Cys loop. (C) The thiyl radical is proposed to abstract a hydrogen atom from the methyl group of toluene. The resulting benzylic group can add to the alkene of fumarate, resulting in a succinate radical which can abstract a hydrogen atom from Cys to ultimately form *R*-benzylsuccinate and regenerate the thiyl radical.^8^^,^^9^^,^^10^ (D) Working model for glycyl radical installation based on X-ray crystal structures. Note that PDB: 5BWE is a structure with glycine, not with the glycyl radical. 1 depicts the crystal structure of BSSαγ (PDB: 5BWD), which contains a partially open, though still buried, glycyl radical domain (GRD, dark gray) as compared to the crystal structure of BSSαβγ (4). It is proposed that this slightly open conformation of BSSαγ (1) could be in equilibrium with a fully open conformation of the GRD (2). The fully open conformation of BSSαγ allows binding of the activating enzyme (AE, green) to the GRD. Once bound, the AE can install the glycyl radical cofactor, thus activating BSS for catalysis (2-> 3). The AE can dissociate and BSSβ can bind to the BSSαγ complex to stabilize the closed conformation (4). It is proposed that a closed conformation of the enzyme is required for toluene binding and fumarate addition.

In oxic environments, hydrocarbons are activated by enzymes that use iron, copper, and flavin cofactors to oxygenate using O_2_.^4^ When molecular oxygen is not present, other cofactors must be used to initiate radical chemistry for hydrocarbon activation. The XSS enzymes use a simple glycyl radical cofactor to initiate radical-based catalysis, which makes them members of the large glycyl radical enzyme (GRE) superfamily.^5^ Members of the GRE superfamily are structurally comprised of a 10-stranded β/α barrel with Gly and Cys loops located within the barrel (Figure 1A). The only structurally characterized XSS to date is benzylsuccinate synthase (BSS), which catalyzes the formation of benzylsuccinate from fumarate and toluene.^6^^,^^7^ Once a radical is formed on the Gly residue within the Gly loop of BSS, it can form a transient thiyl radical on the neighboring Cys residue within the Cys loop (Figure 1B). The thiyl radical selectively abstracts a hydrogen atom from the methyl group of toluene to form a benzylic radical, which can add to the alkene of fumarate in a Giese-like reaction. The newly-formed succinate radical can abstract a hydrogen atom from Cys to form a single enantiomer, *R*-benzylsuccinate (Figure 1C).^8^^,^^9^^,^^10^ The radicals involved in this mechanism, including the glycyl and thiyl radicals, are thought to be protected from off-cycle reactivity by the barrel structure of the GRE.

In the currently available structural data for GREs, if the Gly loop is able to be modeled into electron density, the Gly and Cys loops are close in proximity to one another deep within the GRE barrel (Figure 1A); however, this “closed” conformation of GREs cannot be the sole GRE conformation, as it does not allow for glycyl radical installation to be accomplished. Based on structural and biochemical data, it is thought that a small domain on the C-terminus of the GRE called the glycyl radical domain (GRD) must flip out of the barrel and interact with a partner protein known as an *S*-adenosyl methionine (AdoMet) dependent activating enzyme (AE) (Figures 1D and 1D1–1D3).^6^^,^^11^^,^^12^^,^^13^ Only one GRE-AE has been structurally characterized to date, known as pyruvate formate lyase (PFL)-AE.^14^ PFL-AE contains an active site [4Fe–4S] cluster coordinated by 3 Cys residues.^12^ The unique Fe site can coordinate an equivalent of AdoMet.^15^^,^^16^ On one-electron reduction of the [4Fe–4S]^2+^ cluster to a [4Fe–4S]^1+^ cluster, the C–S bond of AdoMet can be homolytically cleaved, thus forming a 5′-deoxyadenosyl radical. The exact mechanism is an active topic of research,^17^ but canonically it is thought that this radical can abstract an H-atom from the substrate, in this case the Gly residue of the GRE. Based on the peptide-bound crystal structure of PFL-AE, the Gly residue is positioned well for this H-atom transfer to occur; however, there is no way to dock this peptide onto the full “closed” GRE structure without severe steric clashes.^14^^,^^18^^,^^19^ Thus, it is proposed that “open” conformations must exist of GREs in which the Gly loop moves out of the GRE active site and into the GRE-AE active site (Figures 1D, 1D2, and 1D3).^11^^,^^14^ Once glycyl radical is installed, it must move back into the GRE barrel, where it can catalyze multiple rounds of turnover.

Though most characterized GREs consist of a single protein subunit, there are a few exceptions that contain additional smaller subunits, including 4-hydroxyphenylacetate decarboxylase (HPAD)^20^ and BSS.^21^ HPAD contains one additional 9.5 kDa subunit, which harbors two [4Fe–4S] clusters.^22^ BSS contains two extra subunits – BSSγ, which is 7 kDa, and BSSβ, which is 9 kDa, in addition to the 98 kDa large catalytic subunit BSSα.^23^ Like the extra subunit of HPAD, both BSSγ and BSSβ contain [4Fe–4S] clusters.^24^ BSSα cannot be solubly expressed in *Escherichia coli* without BSSγ.^23^ The structure of BSSαγ shows that part of BSSγ fits into a hydrophobic surface pocket of BSSα, which is most likely the reason for increased solubility when the two subunits are coexpressed.^6^*In vivo*, it is known that BSSγ is necessary for organism survival on toluene,^25^ but the native function of this subunit and its [4Fe–4S] cluster is unknown. When comparing the structures of BSSαγ and BSSαβγ, it was observed that the Gly loop of BSSαγ moves out of the active site by 2 Å and the protein begins to partially open in a clam-shell like motion (Figure S2).^6^ It is proposed that the BSSαγ structure has captured a “partially open” snapshot of BSS, where the Gly loop is starting to move out of the GRE active site (Figure 1D1). Based on these movements, BSSβ is proposed to play a role in the conformational changes needed to move between the “closed” state where the Gly loop is near the Cys loop (Figures 1D–1D4) and the “open” state where the Gly loop moves out of the active site completely so it can bind to the GRE-AE for cofactor installation (Figures 1D2 and 1D3).^6^^,^^7^

Although this working model for BSSβ′s role in activation fits the structural data (Figure 1D), it has not been explored biochemically because of the inability to install the glycyl radical within BSS *in vitro*. Multiple attempts have been made to express BSS-AE from *Thauera aromatica* (BSS-AE_Ta_) as soluble protein in *E. coli*, but only very small amounts of folded protein could be obtained, even when expressed as fusion proteins.^7^^,^^26^ Activation experiments have shown that these small amounts of BSS-AE are not able to install glycyl radical on BSS.^26^ This problem with *in vitro* activation has not only hampered our understanding of XSS mechanism, which is unique among the GREs in that it includes multiple subunits, but it has also severely limited the amount of mutagenesis experiments and engineering efforts using these enzymes. A robust system for glycyl radical cofactor installation would open the door to exploring members of this environmentally and synthetically important enzyme class to gain fundamental insight into molecular mechanism and to engineering green, selective biocatalysts for organic synthesis.

Ever since the discovery of BSS in the 1990’s,^27^^,^^28^ the radical chemistry used to carry out this challenging olefin hydroalkylation reaction has been the topic of multiple reports; however, the inability to install the radical cofactor *in vitro* limited the types of experiments that could be accomplished with this system. Here, we have found an XSS-AE that can be recombinantly produced in *E. coli* and used to form glycyl radical on its native XSS *in vitro*. Moreover, we have shown that it has cross-reactivity with BSS. This cross-reactivity is atypical for the GRE superfamily, as GRE-AEs are typically observed to be highly specific for their native GRE partner.^12^^,^^29^ We have also gleaned important insights about the roles of XSS subunits in glycyl radical formation subsequent fumarate hydroalkylation. These studies not only complement previous structural data in determining the function for BSSβ, but also serve as a starting point for future biochemical investigations of XSS enzymes and directed evolution campaigns. This is enabling technology that will allow the larger community to more rapidly mutate XSS enzymes to both understand how hydrocarbons are anaerobically degraded and to engineer this class for asymmetric C–C bond formation.

## Results

### Finding an XSS-AE that can be produced in *E. coli*

**Figure 2:**
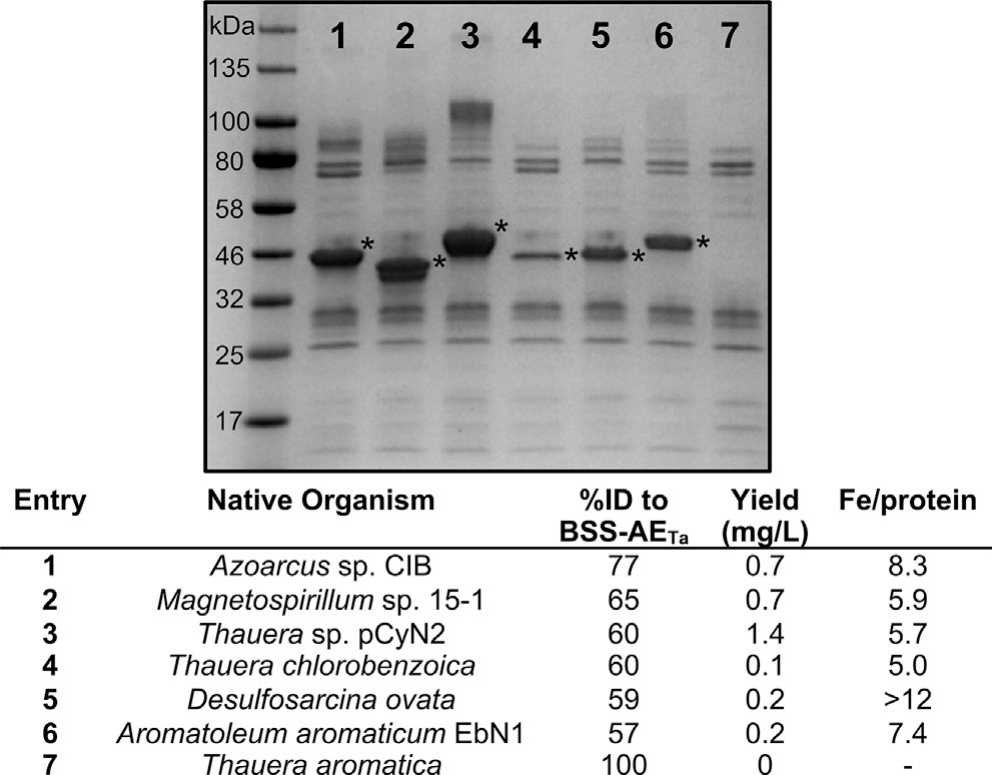
Initial screen of XSS-AEs for solubility SDS-PAGE gel of BSS-AE_Ta_ and 6 XSS-AE homologs after anaerobic immobilized metal affinity chromatography (IMAC) purification. Lane numbers correspond to entry numbers in the table below. Varying amounts of soluble XSS-AEs were observed for the homologs (asterisks denote bands corresponding to XSS-AEs); however, no soluble protein was observed for BSS-AE_Ta_. Impurities in samples were observed because of the low yields of protein obtained in this initial solubility screen; however, upon optimization, pure XSS-AE can be obtained. For each XSS-AE, the native organism, % sequence identity to BSS-AE_Ta_, yield of semi-purified protein, and Fe/protein are reported.

**Figure 3:**
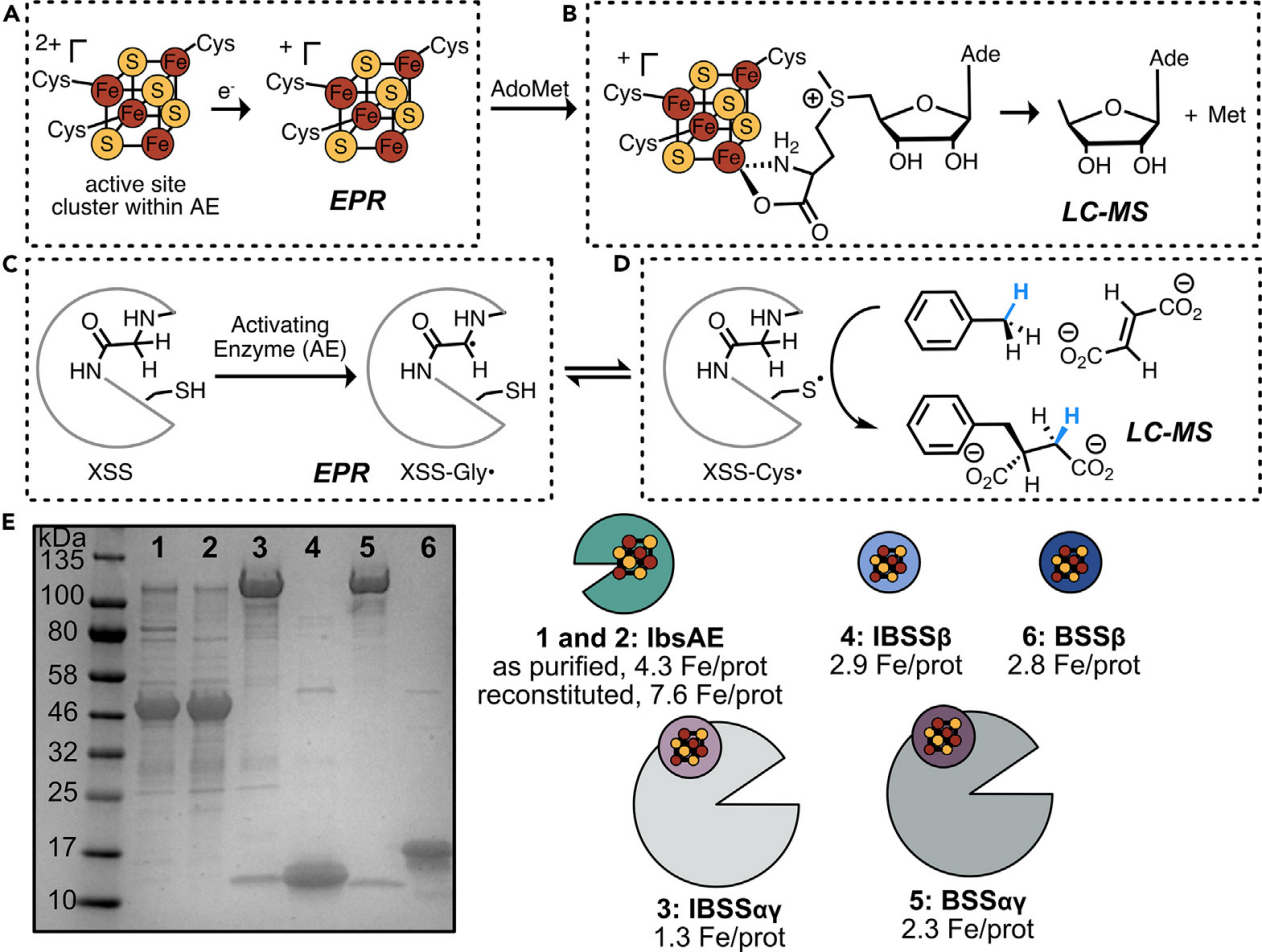
Overview of transformations monitored (A–D) and constructs used (E) in these studies (A) The active site [4Fe–4S] cluster of IbsAE is reduced before catalyzing coupled AdoMet cleavage/glycyl radical installation. (B) The active site [4Fe–4S]^+^ cluster can coordinate an equivalent of AdoMet and reductively cleave it to form methionine and 5′-deoxyadenosine (dAdo). (C) If IbsAE is bound to its partner XSS when AdoMet is reductively cleaved, the essential Gly residue in the XSS can be converted to a glycyl radical through H-atom abstraction by the intermediate 5′-deoxyadenosyl radical. (D) The glycyl radical of the XSS can form a transient thiyl radical on a neighboring Cys residue. This thiyl radical initiates hydrocarbon (e.g. toluene) addition to fumarate. (E) SDS-PAGE gel of purified proteins used in these studies. Lanes 1 and 2 denote the two IbsAE enzymes, “as purified IbsAE” which has not been reconstituted with iron and sulfide and “reconstituted IbsAE.” MW of IbsAE is 40.3 kDa. Lanes 3 and 5 correspond to the IBSSαγ and BSSαγ complexes, respectively. MWs of IBSSα and BSSα are 98.6 and 99.0 kDa, respectively. MWs of IBSSγ and BSSγ are 6.7 and 6.9 kDa, respectively. IBSSβ and BSSβ were purified as separate constructs, not in complex with the IBSSα and BSSα subunits. Lanes 4 and 6 correspond to IBSSβ and BSSβ after affinity tag removal, and their MWs are 8.3 and 9.2 kDa, respectively.

Many gene clusters containing putative XSSs have been discovered and sequenced from anaerobic organisms that are capable of degrading aromatic hydrocarbons, such as toluene. Given the reported insolubility of BSS-AE_Ta_,^7^^,^^26^ we wondered whether recalcitrance to heterologous expression in *E. coli* was a hallmark of XSS-AEs, or whether unexplored homologs could be more easily obtained as pure enzyme. We performed a BLAST search using BSS-AE_Ta_ as a query sequence and chose 6 putative XSS-AEs with different sequences (see Percent Identity Matrix in Table S1) from different organisms (Figure 2, Table S2 and Figure S1). These 6 XSS-AEs, along with BSS-AE_Ta_, were cloned into a pET28a vector that included a C-terminal His_6_-tag. Like all AdoMet-dependent enzymes, the XSS-AEs were predicted to have 3 Cys residues that coordinate an active site [4Fe–4S] cluster (Figure S3). In addition, 8 other Cys residues are conserved within two CX_2_CX_2_CX_3_C motifs, leading us to hypothesize the existence of an additional 2 auxiliary [4Fe–4S] clusters (Figure S3). The domain harboring these auxiliary clusters has been reported for many GRE-AEs; however, its function is still largely unknown. We anaerobically expressed and purified the 7 XSS-AEs in parallel. In accordance with previous reports,^7^^,^^26^BSS-AE_Ta_ was found exclusively in inclusion bodies, with no observable soluble protein (Figure 2, Lane 7). However, the BSS-AE homologs all produced some amount of soluble enzyme, although the range varied considerably, from 0.1 to 1.4 mg of protein per L of culture (Figure 2). The amount of iron in elution fractions was analyzed to estimate how many clusters each of the homologs contained. Five XSS-AEs contained between 5 and 8.3 Fe/protein (Figure 2), which is consistent with these AEs purifying with 1–2 [4Fe–4S] clusters. One homolog, 5, contained more than 12 Fe/protein, which would mean more than 3 [4Fe–4S] clusters could be present. Based on the Cys content of 5, this seems unlikely. It seems more plausible that experimental error in this preliminary solubility screen could be inflating this number. For these studies, instead of following up on 5, which was low yielding, we wanted to continue investigation of the highest yielding homolog, 3, using electron paramagnetic resonance (EPR) spectroscopy and LC-MS (Figures 3A and 3B). This particular XSS-AE, known as 4-isopropylbenzylsuccinate synthase AE (IbsAE), is from a strain of *Thauera* that is known to degrade *p*-cymene.^30^^,^^31^ On optimization of expression conditions for IbsAE, the protein yield was increased to 6 mg/L. Iron content was consistent with one [4Fe–4S] cluster (4.3 Fe/protein, Figure 3E). To determine whether full reconstitution of all 3 [4Fe–4S] clusters could be accomplished, we tried reconstituting the remaining 2 clusters *in vitro*. Similar to previous reports on similar AEs,^32^ full reconstitution was not observed (7.6 Fe/protein, Figure 3E). In addition, 85–90% of the protein precipitated or aggregated as a result of reconstitution (Figure S4).

### IbsAE characterization via EPR spectroscopy and AdoMet cleavage assays

**Figure 4:**
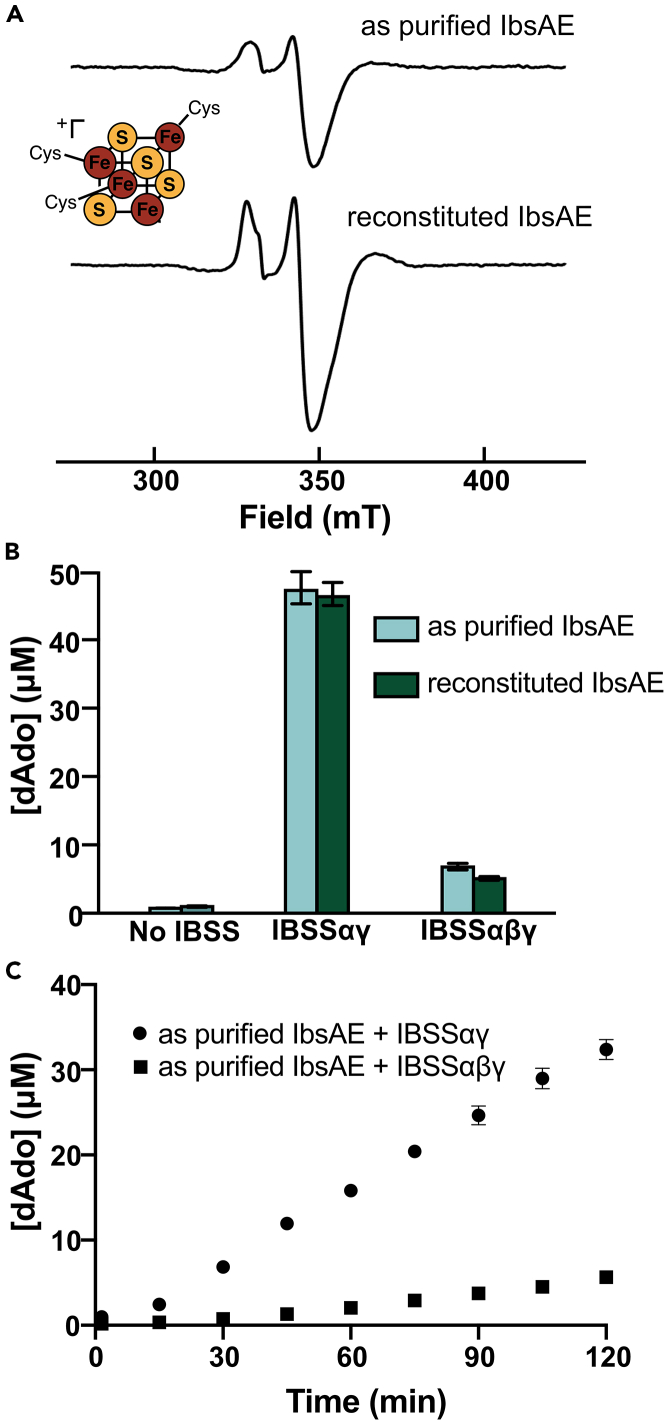
Reduction of the FeS clusters in IbsAE and AdoMet cleavage assays (A) EPR spectra of IbsAE before (as purified) and after (reconstituted) reconstitution of FeS clusters. IbsAE was incubated with dithionite (1 mM) for 1 h prior to freezing. The primary signal observed in both samples is consistent with a [4Fe–4S]^1+^. Conditions of measurement: T = 10 K; microwave power = 50 μW; microwave frequency = 9.37 GHz; modulation amplitude = 10 G; [IbsAE] = 60 μM. (B) AdoMet cleavage by IbsAE was quantified by measuring formation of dAdo by LCMS (n = 3). Trace amounts of dAdo were observed in controls without IbsAE (shown in Table S3). (C) Time course monitoring AdoMet cleavage by IbsAE as measured by LCMS in the presence of either IBSSαγ or IBSSαβγ (n = 3).

Our overarching goal was to produce high enough yields of an XSS-AE to determine conditions suitable for *in vitro* glycyl radical installation and hydroalkylation assays (Figures 3C and 3D). With this goal in mind, we wanted to know if we could use IbsAE as purified instead of reconstituting, as initial attempts to optimize the reconstitution still led to dramatic losses in yield (Figure S4). Based on previous work with other GRE-AEs, we hypothesized that the 4.3 Fe/prot we see in IbsAE corresponds to the active site [4Fe–4S] cluster. To investigate the differences between the ‘IbsAE as purified’ and ‘IbsAE reconstituted’, we turned to EPR spectroscopy. We used 5-deazariboflavin as a photoreductant, which is commonly used for GRE activation assays, to reduce the [4Fe–4S]^2+^ cluster(s). We observed a signal consistent with a [4Fe–4S]^1+^ cluster for the as purified IbsAE (Figure S5). On reduction of the reconstituted IbsAE, we observe a mixture of signals, most likely corresponding to a [4Fe–4S]^1+^ cluster and [3Fe–4S]^1+^ cluster (Figure S5). We reasoned that a stronger reductant may be necessary to reduce the [3Fe–4S]^1+^ cluster to the EPR silent state. When we reduce the as purified and reconstituted IbsAE with dithionite, we see signal for [4Fe–4S]^1+^ cluster without interfering [3Fe–4S]^1+^ cluster signal (Figure 4A, g-values: 1.94 and 2.01). Comparing the double integrals for the two spectra shows that the reconstituted IbsAE contains approximately double the amount of [4Fe–4S]^1+^ cluster, consistent with our iron quantification. Temperature studies corroborate assignment of the signal as [4Fe–4S]^1+^ clusters, where the signal decreases as temperature is increased from 10K to 40K (Figure S6).

After verifying that we did indeed have [4Fe–4S] clusters in both IbsAE as purified and IbsAE reconstituted and that we could reduce these clusters, we assessed the enzymes’ ability to cleave AdoMet in the presence and absence of the corresponding XSS, 4-isopropylbenzylsuccinate synthase (IBSS) (Figure 3B). When we incubate IbsAE with AdoMet following reduction of the [4Fe–4S] cluster, we do observe AdoMet cleavage (Figure 4B, 1.1 μM dAdo), whereas none is observed in the control without IbsAE enzyme (Table S3). Reconstitution of the auxiliary clusters in IbsAE does not affect AdoMet cleavage under these conditions (Figure 4B, 1.1 μM dAdo with as purified IbsAE and 1.4 μM dAdo with reconstituted IbsAE). Oftentimes, low levels of AdoMet cleavage are observed without the substrate bound to IbsAE, as in this particular case; however, typically AdoMet cleavage is accelerated by addition of substrate. In this case, the substrate for IbsAE is the protein complex IBSS. Like BSS, IBSS contains two additional [4Fe–4S]-containing subunits (IBSSβ corresponds to BSSβ and IBSSγ corresponds to BSSγ) in addition to the catalytic IBSSα subunit that harbors the glycyl radical. We obtained the genes for IBSSαβγ with the chaperone protein IbsE. Previous structural and proteolytic data have led to the hypothesis that BSSβ could play an important role in regulating the large conformational changes that must occur to make the catalytic glycine residue physically available to BSS-AE (Figure 1D).^6^ For this reason, we wanted to be able to control the amount of the β subunit that we add to assays. We purified IBSSαγ as a single complex, as previous studies had demonstrated the α subunit does not solubly express without the γ subunit.^23^ We separately expressed and purified IBSSβ and subsequently removed the N-terminal His tag with a TEV cleavage site. Repeating these assays with addition of IBSSαγ does lead to a large increase in AdoMet cleavage (Figure 4B, 47.6 μM dAdo with as purified IbsAE and 46.7 μM dAdo with reconstituted IbsAE). As expected based on our working model (Figure 1D), the addition of IBSSβ with IBSSαγ yields less dAdo product in endpoint assays (Figure 4B, 7.2 μM dAdo with as purified IbsAE and 5.4 μM dAdo with reconstituted IbsAE). The rate of AdoMet cleavage by IbsAE is also slower when IBSSβ is present (Figure 4C and Table S4).

### Glycyl radical formation on IBSS

**Figure 5:**
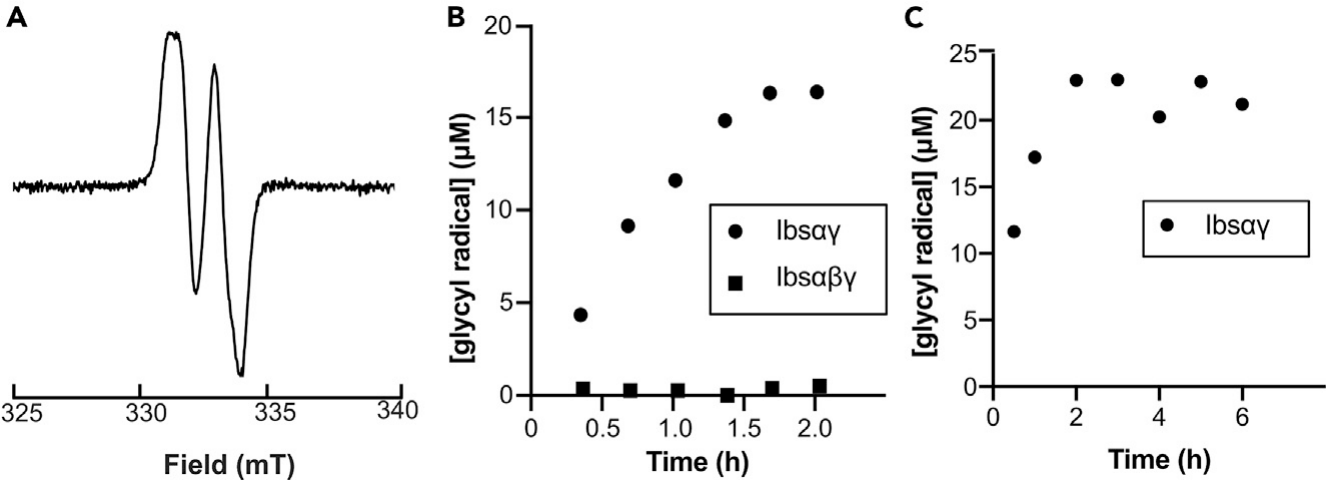
Glycyl radical can be installed in IBSS by IbsAE, thus activating IBSS for catalysis (A–C) (A) Representative EPR spectrum for the glycyl radical in IBSSαγ. Conditions of measurement: T = 80 K; microwave power = 1.26 μW; microwave frequency = 9.37 GHz; modulation amplitude = 3 G; [IBSSαγ] = 50 μM; [IbsAE] = 50 μM. Activation reactions were conducted for different lengths of time and frozen for EPR analysis. Double integrals of EPR spectra were calculated using Xenon software and compared to double integrals of known concentrations of Fremy’s salt standards to calculate concentration of radical in μM. These concentrations of radical were plotted versus time to produce plots (B) (comparison of radical installation in IBSSαγ versus IBSSαβγ) and (C) (6 h time course of radical installation in IBSSαγ).

Following validation that IbsAE is able to cleave AdoMet, we next wanted to determine whether glycyl radical within IBSS could be observed by EPR spectroscopy (Figure 3C). We tried activating IBSSαγ with and without IBSSβ using our as purified IbsAE stock, which is missing its auxiliary [4Fe–4S] clusters. Consistent with AdoMet cleavage assays as well as our working model, we only observe significant quantities of glycyl radical without IBSSβ (Figures 5A and 5B and Table S5). The observation that IbsAE is able to form a glycyl radical on IBSSαγ without full reconstitution of its auxiliary [4Fe–4S] clusters is consistent with work showing that 4-Hpad-AE can also activate its corresponding GRE without the auxiliary clusters.^33^ However, the persistence of the glycyl radical was significantly affected in previous studies of 4-Hpad-AE, and within 16 min, most of the radical was gone.^33^ Time courses of activation reactions with our as purified IbsAE demonstrate that radical persistence is not an issue with this system for at least up to 6 h (Figure 5C and Table S6). It was also found that the as purified IbsAE installed glycyl radical within IBSSαγ faster than the reconstituted IbsAE (Figure S7), which is convenient given our low yields of reconstituted IbsAE.

### Cross-reactivity is observed for glycyl radical installation on BSS

**Figure 6:**
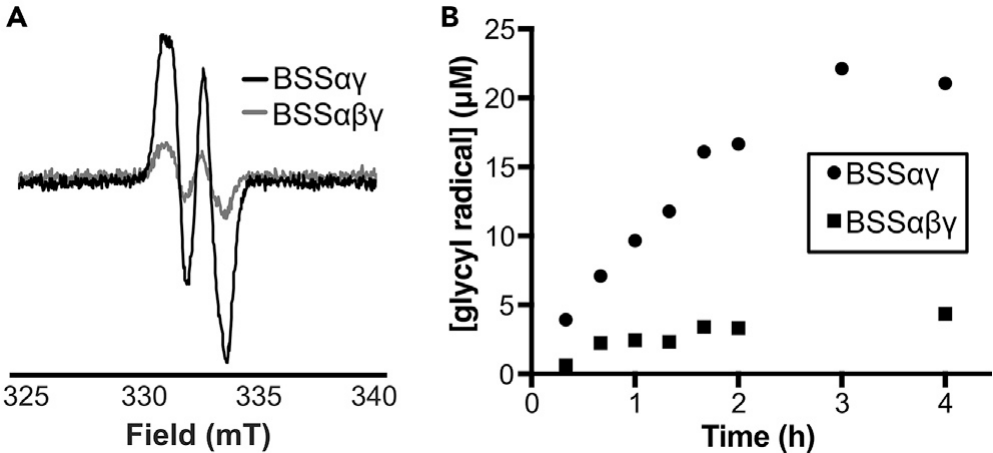
Cross-reactivity is observed between IbsAE and BSS (A and B) (A) Representative EPR spectrum for the glycyl radical in BSSαγ (black) and BSSαβγ (gray). Conditions of measurement: T = 80 K; microwave power = 1.26 μW; microwave frequency = 9.37 GHz; modulation amplitude = 3 G; [BSSαγ] = 50 μM; [IbsAE] = 50 μM. Activation reactions were conducted for different lengths of time and frozen for EPR analysis. Double integrals of EPR spectra were calculated using Xenon software and compared to double integrals of known concentrations of Fremy’s salt standards to calculate concentration of radical in μM. These concentrations of radical were plotted versus time to produce plot (B), comparing radical installation in BSSαγ versus BSSαβγ.

To date, BSS remains the only structurally characterized XSS, and significantly more is known about the scope and mechanism for this enzyme than XSSs that function on substrates beyond toluene (e.g. IBSS). We wondered whether IbsAE could activate BSS as well. Although GRE-AEs are typically highly specific for their partner GRE,^12^^,^^23^^,^^29^ we do observe an EPR signal consistent with the glycyl radical when BSSαγ is incubated with reduced IbsAE and AdoMet (Figure 6A). We wanted to test the effects of BSSβ on glycyl radical installation as well as the persistence of radical on BSSαγ. Activation time courses were performed for BSSαγ and BSSαβγ over the course of 4 h. Similar to IBSS, less radical is formed when BSSβ is added to the reactions. Similar amounts of radical are formed on BSSαγ as IBSSαγ, and this radical persists for the timescale of the experiment (Figure 6B and Table S7).

### BSSβ is necessary for high benzylsuccinate production

**Figure 7:**
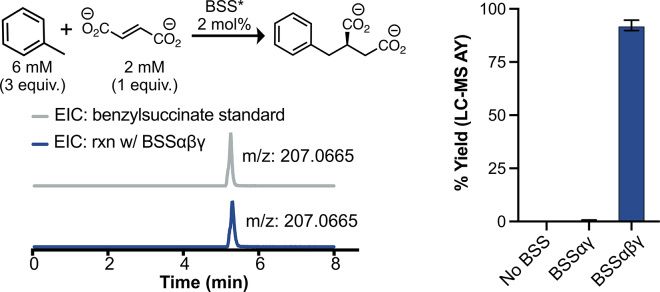
Activated BSSαβγ can catalyze the addition of toluene to fumarate High resolution LCMS was used to monitor formation of benzylsuccinate. A standard curve was prepared for benzylsuccinate using commercially available benzylsuccinate and L-tryptophan as an internal standard. Assay yields were determined by integrating the EIC spectrum for benzylsuccinate and calculating yield using the standard curve, with fumarate as the limiting reagent (n = 9).

After demonstrating that BSSαγ can be activated, we wanted to determine whether we could observe hydroalkylation activity *in vitro*. We activated BSSαγ for 3 h and subsequently added fumarate (2 mM final conc.) and toluene (6 mM added as a solution of toluene and MeOH). BSSβ was added to some reactions to test the effects of this subunit on hydroalkylation yields. We detected and quantified product formation using high-resolution QToF-LCMS. Nine replicates of each reaction condition were conducted in parallel. In reactions with BSSαγ and BSSαβγ, a peak with the same retention time and the same exact mass as a benzylsuccinate (BS) authentic standard was observed (Figure 7). Yields increased dramatically in the presence of BSSβ (Figure 7 and Table S8, from 0.7% to 92.3% assay yield). Control reactions without BSSαγ produced no detectable BS in 7 of 9 samples and trace levels of BS in 2 of 9 samples (Figure 7 and Table S8).

## Discussion

In this work, we set out to solve the long-standing problem of *in vitro* glycyl radical cofactor installation in BSS. Having an *in vitro* system is useful for both probing the molecular mechanism of XSSs as well as for developing XSSs as biocatalysts. XSSs are crucial to anaerobic hydrocarbon degradation within microbes, and thus understanding how these enzymes work within their native cellular environments remains an important question. By developing an *in vitro* activation method, we were able to explore the molecular mechanism of BSS activation and catalysis in ways that were not previously possible. Prior biochemical investigations and crystal structures showed that BSS contains two accessory subunits, each with a [4Fe–4S] cluster bound.^6^^,^^23^ Here, we explore hypotheses regarding the function of one of these subunits – BSSβ. Based on crystallographic data, we previously proposed that BSSβ is needed to control conformational dynamics of the glycyl radical domain and plug the hydrocarbon substrate channel.^6^^,^^7^ XSSs have to position hydrocarbon substrates for radical catalysis and, unlike substrates of single-component GREs, XSS substrates have no functional handles to help control positioning. Moreover, GREs are in equilibrium between an “open” state, where GRE-AE can install glycyl radical, and a “closed” state, where catalysis can occur. It is proposed that changing conditions, such as GRE-AE concentration, can shift this equilibrium.^6^^,^^11^ Our hypothesis was that BSSβ binding shifts the equilibrium of BSS to the closed state, which allows for tighter control over hydrocarbon binding. Based on this model, we would expect two clear observations: (1) when BSSβ is present, AE should not be able to activate BSS as well, and (2) BSSβ should be necessary for catalysis. We wanted to test this hypothesis biochemically, but we could not obtain soluble BSS-AE. Here, we use genome mining to find a soluble XSS-AE with cross-reactivity for BSS. When we tested the role of BSSβ on activation and catalysis, we do indeed observe that BSSβ inhibits activation and is necessary for catalysis.

Many more questions remain about the molecular details of XSS activation and hydroalkylation activity, which can now be more readily probed. For example, the roles of the metalloclusters within the accessory subunits are still poorly understood. In BSSγ, when the [4Fe–4S] cluster is removed (either through metal chelation or mutagenesis), it appears to dissociate from BSSα.^23^ BSSγ binding is known to enhance BSSα solubility when heterologously expressed in *E. coli*. The current data point to a model where the [4Fe–4S] cluster is necessary for BSSγ to adopt a conformation to bind BSSα, and this binding is necessary to plug a hydrophobic patch on BSSα for solubility. We are still unsure if this is BSSγ′s native role, or if these are artifacts of heterologous expression. Moreover, there is no proposed role for BSSβ′s [4Fe–4S] cluster. Marsh et al. showed that they can create a mutant of BSSβ that does not bind iron but were not able to assess its effects on activation or catalysis.^23^ Using our *in vitro* system, these experiments could be revisited to determine the role of BSSβ′s [4Fe–4S] cluster. Beyond arylalkyl-succinate synthases like BSS, there are numerous alkyl-succinate synthases that functionalize saturated hydrocarbons. Even less is known about these enzymes that can directly and selectively functionalize saturated alkanes; for example, the subunit/cofactor architecture of these enzymes is still unknown and is thought to include an additional subunit.^34^ Could we use a similar genome mining approach to find soluble alkyl-SS-AEs as well?

XSSs also hold the potential to be useful synthetic tools for building small molecules. They use abundant feedstocks (hydrocarbons and olefins) to form new Csp^3^–Csp^3^ bonds using radical hydroalkylation. Hydroalkylation chemistry is an attractive method for forming C–C bonds as it has the potential to set multiple stereocenters at once; however, controlling the stereoselectivity remains challenging. Inside radical enzymes, substrate positioning can provide control over stereoselectivity, for example, toluene addition to fumarate to form exclusively *R*-benzylsuccinate in BSS. Beyond BSS, other XSSs exist that are able to perform this chemistry using a wide range of hydrocarbons, including saturated hydrocarbons. Given that the transformations they catalyze could prove so useful, why have XSSs not been widely explored as biocatalysts? One major hurdle has been activating the glycyl radical cofactor. Until now, activation has only been accomplished in whole cells. Purification of activated BSS from whole cells results in rapid loss of hydroalkylation activity.^23^ The inability to generate pure, activated BSS has limited the types of studies that can be done, including characterization of variants made through mutagenesis. Recently, other groups have also developed tools to circumvent these issues. In 2021, Heider et al. developed a heterologous expression and activation system in *Aromatoleum evansii*, which importantly cannot degrade toluene, to assess BSS variants.^26^ In addition to providing insight into the mechanism of substrate recognition, Heider et al. showed that the olefin substrate is not restricted to dicarboxylic acids.^26^ Even more recently, Cirino et al. developed a heterologous system for producing alkylsuccinates in *E. coli* using a BSS homolog, allowing even more rapid access to substrate scope studies.^34^ Although these tools will accelerate the development of XSSs as biocatalysts, a key limitation still existed – *in vitro* activation and subsequent hydroalkylation using purified enzymes. Whole cell activation for screening of XSS variants is attractive from a high-throughput standpoint. However, with the ability to activate *in vitro*, we now can conduct reactions using purified enzymes to verify findings from whole cell screening and rationalize the effects of key mutations. By combining approaches, mutagenesis studies can much more rapidly be accomplished for this enzyme class.

XSSs have fascinated and challenged scientists for decades, and numerous studies^5^^,^^6^^,^^7^^,^^21^^,^^23^^,^^24^^,^^25^^,^^26^^,^^27^^,^^28^^,^^34^ have helped to shed light on their mechanism. Methods developments reported here and described above are likely to rapidly accelerate our understanding of XSS enzyme mechanisms, XSS substrate scope, and enable XSS protein engineering efforts. We are excited to see how these future efforts will reshape the way we view XSSs and what we understand about them.

### Limitations of the study

This study focuses on the development of methodology for studying XSSs but does not explore substrate scope for these enzymes. Also, it was shown that reconstitution of auxiliary clusters within the ferredoxin domain of IbsAE is not necessary for enzyme activity, but the function of this domain and its clusters was not thoroughly investigated. In addition, in Figure 2, initial yields and Fe content of XSS-AE homologs were calculated using the protein fractions shown in the gel in Figure 2, which do contain impurities that affect the accuracy of the calculated yield and Fe content. Because the results in Figure 2 were meant to serve as an initial screen to determine which homolog was the highest yielding, expression and purification of only IbsAE was optimized for improved yield and purity.

## STAR★Methods

### Key resources table

**Table:** 

REAGENT or RESOURCE	SOURCE	IDENTIFIER
**Bacterial and virus strains**		

*E.* coli DH5α cells	New England BioLabs	C2987H
*E.* coli T7 Express cells	New England BioLabs	C2566H

**Chemicals, peptides, and recombinant proteins**		

Benzylsuccinate	Sigma Aldrich	CAS 884-33-3
potassium nitrosodisulfonate	Sigma Aldrich	CAS 14293-70-0
*S*-Adenosyl methionine	Sigma Aldrich	CAS 86867-01-8
iron standard	Alfa Aesar	EINECS 231-714-2
Ferene	Sigma Aldrich	CAS 79551-14-7

**Oligonucleotides**		

All primers used are reported in “method details” and Table S2	Sigma Aldrich	N/A

**Recombinant DNA**		

All purchased XSS-AE genes are reported in Table S2	Twist Biosciences	N/A
XSS genes are reported in “method details”	Twist Biosciences	N/A
BSS-AE_Ta_ gene	Ref #^23^, Marsh lab	N/A

**Software and algorithms**		

Xenon Software	Bruker	N/A
Clustal2.1	Ref #^1^ in supplemental information	N/A
MassHunter Software	Agilent	N/A

### Resource availability

#### Lead contact

Further information and requests for resources and reagents should be directed to and will be fulfilled by the lead contact, Catherine L. Drennan (cdrennan@mit.edu).

#### Materials availability

All unique plasmids generated in this study are available from the lead contact without restriction. No unique reagents were generated.

### Experimental model and study participant details

Cell lines used in this study, *E.* coli DH5α cells and T7 Express cells, were purchased from New England BioLabs. Growth conditions are reported in the method details section below.

### Method details

#### Construction of expression plasmids

##### BSS-AE_Ta_ homologs

The genes for the six BSS-AE_Ta_ homologs were identified through a BLAST search using the amino acid sequence of BSS-AE_Ta_. Results were narrowed down based on literature precedent (i.e. the native organism had been characterized as an anaerobic aromatic hydrocarbon degrader) and sequence similarity (i.e. genes with very high similarity to one another were excluded, see Table S1). The amino acid sequences for the 6 genes (see Table S1 for gene identifiers) were used for codon optimization for expression in *E. coli* K12. The six genes were purchased from Twist Bioscience, where they were cloned into pET28a at restriction sites BamHI and HindIII. The resulting plasmids contained both N-terminal and C-terminal hexa-His-tags. The N-terminal His-tag was removed from all AE constructs using Q5® Site-Directed Mutagenesis Kit (New England Biolabs) using the primers in Table S2. All primers were designed using NEBaseChanger™. All genes were confirmed through Sanger sequencing by Quintara Biosciences.

##### BSS-AE_Ta_

We received the gene for BSS-AE_Ta_ from the Marsh lab.^23^ The BSS-AE_Ta_ gene was amplified and overhangs were added with complementarity to pET28a using the following primers: forward primer, 5′- CTTTAAGAAGGAGATATACCATGAAAATTCCATTAGTCAC-3′ and reverse primer, 5′-TCGAGTGCGGCCGCAAGCTTCCTTTTCGGGTGGGTCTCTT-3′. The pET28a vector was amplified and overhangs were added with complementarity to BSS-AE_Ta_ using the following primers: forward primer, 5′- AAGAGACCCACCCGAAAAGGAAGCTTGCGGCCGCACTCGA-3′ and reverse primer, 5′- GTGACTAATGGAATTTTCATGGTATATCTCCTTCTTAAAG-3′. PCRs were conducted using the Q5® Site-Directed Mutagenesis Kit (New England Biolabs). PCR products were purified over 1% agarose gels using a Qiagen gel extraction kit. Gibson assembly reactions using NEBuilder® HiFi DNA assembly were set up at 50°C for 1 hour with an insert:vector ratio of 3:1. A small aliquot (5 μL) of the reaction was transformed into *E.* coli DH5α cells (New England BioLabs). The resulting construct was verified through Sanger sequencing by Quintara Biosciences.

##### IBSS and BSS

The plasmids used to express BSSαβγ were published previously. Briefly, BSSα and BSSγ were cloned into a pET-DUET vector into sites NdeI/KpnI and NcoI/HindIII, respectively. BSSα was C-terminally His_6_ tagged. BSSβ and TutH were cloned into a pRSF-DUET plasmid into sites NdeI/XhoI and NcoI/HindIII. For expression of BSSαγ with the TutH chaperone, the β-subunit was removed from the pRSF-DUET plasmid using the Q5® Site-Directed Mutagenesis Kit (New England Biolabs) and the following primers: forward primer, 5′- CTCGAGTCTGGTAAAGAAAC-3′ and reverse primer, 5′- ATTTCGATTATGCGGCCG-3′. His_6_-BSSβ, which included a TEV cleavage site after the N-terminal His_6_ tag, was constructed using the Q5® Site-Directed Mutagenesis Kit (New England Biolabs) and the following primers: forward primer, 5′- AACGACCGAGAATCTTTATTTTCAGGGATCCGAGGGCAGCAACATGGAA-3′ and reverse primer, 5′- GGATCGTGATGGTGATGGTGATGGCTGCTAGCCATATGTATATCTCCTTCTTATACTTAACTAATATAC-3′.

For the IBSS complex, the amino acid sequences (IBSSα - UniProt ID: A0A096ZNX3, IBSSβ - UniProt ID: A0A096ZP03, IBSSγ - UniProt ID: A0A096ZNX6) and putative chaperone protein (IbsE – UniProt ID: A0A096ZNY2) were used for codon optimization for expression in *E. coli* K12. The genes were purchased from Twist Bioscience as linear g-blocks with overhangs for Gibson Assembly. A pET-DUET plasmid was amplified using the Q5® Site-Directed Mutagenesis Kit (New England Biolabs) and the following primers: forward primer, 5′-AGCGCAGCTTAATTAACCT-3′ and reverse primer, 5′-GGTATATCTCCTTCTTAAAGTTAAACAA-3′. PCR product was purified over 1% agarose gels using a Qiagen gel extraction kit. C-terminally His_6_ tagged IBSSα and IBSSγ were assembled into the linearized pET-DUET vector using NEBuilder® HiFi DNA assembly kit. A pRSF-DUET plasmid was amplified using the Q5® Site-Directed Mutagenesis Kit (New England Biolabs) and the following primers: forward primer, 5′-CTCGAGTCTGGTAAAGAAAC-3′ and reverse primer, 5′-CCATGGTATATCTCCTTATTAAAG-3′. PCR product was purified over 1% agarose gels using a Qiagen gel extraction kit. IbsE and IBSSβ were assembled into the linearized pET-RSF vector using NEBuilder® HiFi DNA assembly kit. For expression of IBSSαγ, the IBSSβ was removed from the pRSF-DUET plasmid using the Q5® Site-Directed Mutagenesis Kit (New England Biolabs) and the following primers: forward primer, 5′- CTCGAGTCTGGTAAAGAAAC-3′ and reverse primer, 5′- TTAGACACGCGCTTTTGC-3′. His_6_-IBSSβ, which included a TEV cleavage site after the N-terminal His_6_ tag, was constructed using the Q5® Site-Directed Mutagenesis Kit (New England Biolabs) and the following primers: forward primer, 5′- AACGACCGAGAATCTTTATTTTCAGGGATCCGCTAATGTGCAGACCCAG-3′ and reverse primer, 5′- CAATTCATATTCTTCCTCTATATGTATACCGATCGTCGGTAGTGGTAGTGGTAGTGCTAGG-3′. All BSS and IBSS constructs were verified through Sanger sequencing by Quintara Biosciences.

#### Expression and purification of constructs

##### XSS-AE solubility screen

XSS-AE constructs were transformed into T7 Express cells (New England BioLabs) and a single colony was used to make a glycerol stock of each. Starter cultures were inoculated from glycerol stocks and grown overnight in LB containing 50 μg/mL kanamycin for each XSS-AE at 37°C at 220 rpm. LB media (1L) containing 50 μg/mL kanamycin, 150 mg iron(II) ammonium sulfate hexahydrate (CAS: 783-85-9), and 47 mg L-cysteine was inoculated with 10 mL of starter culture. Expression cultures were grown at 37°C at 220 rpm to an OD600 = 0.8, at which point they were induced with 1 mM IPTG (GoldBio). Induced cultures were expressed for 4 h at 22°C at 100 rpm. Cells were pelleted by centrifugation, flash frozen in liquid nitrogen, and stored at −80°C until lysis. Cell lysis and protein purification were performed anaerobically in an MBraun chamber. All buffers were sparged with argon before use. For lysis of cells, cell paste was resuspended in 15 mL lysis buffer (lysis buffer: 50 mM HEPES pH 8.0, 300 mM NaCl, 2 EDTA-free protease inhibitor pellet (cOmplete, Roche Diagnostics), 100 mg lysozyme (Sigma Aldrich), and 8 μL benzonase (EMD Millipore). Cells were resuspended by mashing cell paste with a spatula. Resuspended cells were incubated for 30 min at 4°C, after which cells were sonicated for a 1 min cycle of 2 s on and 15 s off at an amplitude of 10 (Qsonica). Lysate was clarified by centrifugation for 45 min at 28,000 g and subsequently filtered (0.22 μm) before purification. XSS-AEs were purified in parallel on 0.5 mL of TALON resin, which was gravity-packed into 2 mL plastic spin columns (Thermo Scientific™ Pierce™ Centrifuge Columns). Columns were equilibrated with 10 mL equilibrations buffer (50 mM HEPES pH 8.0, 300 mM NaCl) before passing cell lysate through by gravity. Columns were washed with 10 mL of wash buffer (50 mM HEPES pH 8.0, 300 mM NaCl, 5 mM imidazole) and eluted into new 15 mL falcon tubes with 4 mL of elution buffer (50 mM HEPES pH 8.0, 300 mM NaCl, 100 mM imidazole). Concentration of protein in each eluent was determined using a Bradford assay. Iron quantification was conducted using a ferene assay^35^ (ferene purchased from Sigma Aldrich, CAS 79551-14-7) and iron standards (EINECS 231-714-2).

##### IbsAE large scale expression and purification

After optimization of IbsAE expression conditions, the following protocol was found to yield the highest amounts of IbsAE. Starter cultures were inoculated from glycerol stocks and grown overnight in LB containing 50 μg/mL kanamycin at 37°C at 220 rpm. Expression cultures were inoculated with 10 mL of starter culture per 1 L of TB containing 50 μg/mL kanamycin, 150 mg iron(II) ammonium sulfate hexahydrate (CAS: 783-85-9), and 47 mg L-cysteine. Eight liters total of culture were grown per round of expression and purification, split into 1 L cultures in 2.5 L flasks. Expression cultures were grown at 37°C at 220 rpm to an OD600 = 0.8, at which point they were induced with 1 mM IPTG (GoldBio). Induced cultures were expressed overnight (16–20 h) at 22°C at 100 rpm. Cells were pelleted by centrifugation, flash frozen in liquid nitrogen, and stored at −80°C until lysis. Cell lysis and protein purification were performed anaerobically in an MBraun chamber. All buffers were sparged with argon before use. For lysis of cells, cell paste from 2 L of culture was resuspended in 25 mL lysis buffer (lysis buffer: 50 mM HEPES pH 8.0, 300 mM NaCl), with an EDTA-free protease inhibitor pellet (cOmplete, Roche Diagnostics), lysozyme (1 mg lysozyme/ml buffer, Sigma Aldrich), and 2 μL benzonase (EMD Millipore). Cells were resuspended by mashing cell paste with a spatula. Resuspended cells were incubated for 30 min at 4°C, after which cells were sonicated for 2 × 1 min cycles of 2 s on and 15 s off at an amplitude of 10 (Qsonica). Lysate was clarified by centrifugation for 45 min at 28,000 g and subsequently filtered (0.22 μm) before purification. IbsAE was purified on 6 mL of TALON resin, which was gravity-packed into two 10 mL plastic spin columns (Thermo Scientific™ Pierce™ Centrifuge Columns). Columns were equilibrated with 30 mL equilibration buffer (50 mM HEPES pH 8.0, 300 mM NaCl) before passing cell lysate through by gravity. Columns were washed with 30 mL of wash buffer (50 mM HEPES pH 8.0, 300 mM NaCl, 5 mM imidazole) and eluted into new 50 mL falcon tubes with ∼20 mL of elution buffer (50 mM HEPES pH 8.0, 300 mM NaCl, 100 mM imidazole). IbsAE was buffer exchanged into 50 mM HEPES pH 8.0, 300 mM NaCl, concentrated to ∼300–500 μM, aliquoted and flash frozen. Concentration of protein was determined using a Bradford assay. Iron quantification was conducted using a ferene assay^35^ and iron standards (EINECS 231-714-2).

##### Reconstitution of IbsAE

IbsAE purified with an intact active site cluster following the purification protocol described above. For reconstitution of the auxiliary clusters, ∼2 mL of purified IbsAE (∼100 μM) was reconstituted at a time. IbsAE was thawed in an MBraun chamber at 4°C at which point DTT was added to a final concentration of 10 mM and was incubated for an hour. Five molar equivalents of Fe(III)Cl_3_ were added to the protein, which was immediately mixed. Five molar equivalents of Na_2_S were added to the protein, which was immediately mixed. The reconstitution was allowed to incubate for 30 minutes; then 5 more equivalents of both Fe(III)Cl_3_ and Na_2_S were added as described above. The reconstitution was incubated for 2 more hours, was spun at 14,000 g for 10 minutes, and was filtered (0.22 μm). After filtration, reconstituted IbsAE was purified by size exclusion chromatography on an S200 16/60 column (50 mM HEPES pH 8.0, 300 mM NaCl, 1 mM DTT). The monomer peak was collected, concentrated, and flash frozen.

##### BSS and IBSS large scale expression and purification

All BSS and IBSS constructs were transformed into T7 Express cells (New England BioLabs) and a single colony was used to make a glycerol stock of each. Starter cultures were inoculated from glycerol stocks and grown overnight at 37°C at 220 rpm in LB containing either 50 μg/mL kanamycin and 100 μg/mL ampicillin for IBSSαγ and BSSαγ or 50 μg/mL kanamycin for IBSSβ and BSSβ. Expression cultures were inoculated with 10 mL of starter culture per 1 L of LB containing the corresponding antibiotics, 150 mg iron(II) ammonium sulfate hexahydrate (CAS: 783-85-9), and 47 mg L-cysteine. Expression, TALON purification, and concentration were carried out anaerobically as described above in “IbsAE large scale expression and purification.” For IBSSβ and BSSβ, the N-terminal His-tag was cleaved with His-tagged TEV protease at a ratio of 10:1 (β subunit:TEV protease, w/w). The reaction was gently mixed and left at 4°C for ∼24 hours (or until >80% completion as determined by SDS-PAGE) without agitation. The reaction mixture was purified on TALON resin as detailed above. Fractions containing pure IBSSβ or BSSβ, with the Histag removed, were pooled and buffer exchanged into 50 mM HEPES pH 8.0, 300 mM NaCl.

#### EPR spectroscopy of [4Fe–4S] clusters

##### Reduction of the [4Fe–4S] clusters

In many cases, flavin derivatives are used to reduce the active site cluster of GRE-AEs to initiate glycyl radical installation. Two flavin derivatives, acriflavine^36^ and 5-deazariboflavin,^11^ were tested for their ability to reduce the active site cluster of as purified IbsAE. In a Coy anaerobic chamber, IbsAE (60 μM) was incubated with either acriflavine and bicine (100 μM and 50 mM, respectively) or deazariboflavin (100 μM) in activation buffer (20 mM Tris pH 7.5, 100 mM KCl) for 30 minutes. Most IbsAE precipitated out of solution when acriflavine was added, so it was no longer pursued as a photoreductant. IbsAE remained in solution with deazariboflavin and an EPR signal consistent with a [4Fe–4S]^1+^ cluster was observed. When the protocol using deazariboflavin was used to reduce the reconstituted IbsAE, a mix of signals was observed, corresponding to [4Fe–4S]^1+^ cluster and [3Fe–4S]^1+^ cluster. Reductions for both as purified IbsAE and reconstituted IbsAE were repeated with dithionite (1 mM final concentration) and incubated for an hour. Dithionite-reduced samples produced primarily signals consistent with [4Fe–4S]^1+^ clusters.

##### EPR parameters

EPR spectra were collected in a Bruker EMX-Plus spectrometer at temperatures between 10–40 K with a Bruker/ColdEdge 4K waveguide cryogen-free cryostat. Xenon 1.1b.155 software was used to collect and process spectra. Spectra were recorded at 9.37 GHz with a modulation amplitude of 10 G, microwave power of 50 μW, and a 100 kHz modulation frequency. A center field of 3500 G, a sweep time of 60 s, and a sweep width of 2000 G were used. Each spectrum shown is an average of 10 scans. The double integrals of the two spectra in Figure 4A were calculated using Xenon software and compared to one another to determine the relative amount of [4Fe–4S]^1+^ cluster in each.

#### Activations to install glycyl radical

In a Coy anaerobic chamber, reduction reactions were conducted by combining activation buffer (20 mM Tris pH 7.5, 100 mM KCl), 5-deazariboflavin (200 μM final conc.), DTT (2 mM final conc.), and IbsAE (100 μM final conc.). The reduction was gently mixed and illuminated using an LED light for 30 minutes. The reduction was diluted with activation buffer such that the final concentration of IbsAE was 50 μM. IBSSαγ or BSSαγ (50 μM final conc.), IBSSβ or BSSβ (0 or 50 μM final conc.), and AdoMet (1.5 mM final conc., Sigma Aldrich CAS 86867-01-8) were added and the reaction was gently mixed. Reactions were conducted at room temperature without agitation by the LED lamp for 0.3–6 hours, at which point they were either used in AdoMet cleavage assays or hydroalkylation reactions, or anaerobically frozen in liquid nitrogen for EPR spectroscopy.

#### EPR spectroscopy to quantitate glycyl radical

EPR spectra of the glycyl radical was collected at 80 K. Spectra were recorded at 9.37 GHz with a modulation amplitude of 3 G, microwave power of 1.26 μW, and a 100 kHz modulation frequency. A center field of 3350 G, a sweep time of 21 s, and a sweep width of 200 G were used. Each spectrum shown is an average of 10 scans. Potassium nitrosodisulfonate (Fremy’s salt, Sigma Aldrich) was used as a standard. The double integrals of each spectrum were calculated using Xenon software and compared to the double integrals obtained from Fremy’s standard to obtain concentrations of glycyl radical.

#### LCMS/MS assays

Product formation in AdoMet cleavage and hydroalkylation assays was quantified using a Q-TOF LC/MS (Agilent 6545 mass spectrometer coupled to an Agilent Infinity 1260 liquid chromatography system) and a Zorbax reversed-phase column (300SB-C18, 3.5 μm, 2.1 × 50 mm, Agilent). Solvent A was H_2_O with 0.1% acetic acid, and solvent B was acetonitrile with 0.1% acetic acid. Flow rate was 0.4 mL/min. The LC method for all assays was as follows: 0–2 min, 1% B; 2–4 min, gradient from 1 to 50% B; 4–6 min, gradient from 50 to 100% B; 6–7 min, 100% B; 7–8 min, gradient from 100 to 1% B.

##### AdoMet cleavage

Cluster reductions and AdoMet cleavage reactions were conducted as described above. Endpoint assays were conducted for 2 hours and time courses were conducted for 1.5–120 minutes. Reactions were quenched with one volume of methanol and 100 μM of L-tryptophan was added as an internal standard. Quenched reactions were removed from the Coy and protein was pelleted by centrifugation. The resulting supernatant was filtered through a 0.22 μm filter and used for LC/MS analysis. The LC method described above was used with the MS in positive ion mode. Extracted ion counts for 5′-deoxyadenosine (dAdo) and L-tryptophan were obtained, and the concentration of product was determined using a standard curve made from known amounts of dAdo and L-Trp.

##### Hydroalkylation

Cluster reductions and glycyl radical installation were conducted as described above. BSSβ was not added to glycyl radical installation reactions. Three hours after glycyl radical installation reactions were initiated, fumarate (2 mM final conc.) and toluene (6 mM final conc. added as a stock solution in MeOH, 3% v/v) were added. Reactions were diluted such that the final conc. of BSSαγ was 40 μM, and BSSβ (40 μM final conc.) was also added to some reactions. Control reactions contained all components except BSSαγ. Reactions were conducted in a Coy anaerobic chamber in a 96-well microtiter plate with a final volume of 25 μL for each reaction. Hydroalkylation reactions were quenched with two volumes of methanol and 100 μM of L-tryptophan was added as an internal standard. Quenched reactions were removed from the Coy and protein was pelleted by centrifugation. The resulting supernatant was diluted 20-fold and filtered through a 0.22 μm filter and used for LC/MS analysis. The LC method described above was used with the MS in negative ion mode. The retention times for fumarate, L-Trp, and benzylsuccinate were 1.218, 4.393, and 5.240 min, respectively. Product concentration was determined by the extracted ion count ratio of benzylsuccinate and internal standard L-Trp, multiplied by response factor 0.21, which was established via a calibration curve with known amounts of benzylsuccinate (Sigma Aldrich, CAS 884-33-3) and L-Trp. The assay yield was defined as 100x[BS]/2 mM, where 2 mM represents the initial concentration of the limiting reagent, fumarate.

### Quantification and statistical analysis

In Figure 4B/Table S3 and Figure 4C/Table S4, number of assays conducted for each condition was equal to 3 (n = 3), and the mean and the standard deviations were calculated in Excel. In Figure 7/Table S8, number of assays conducted for each condition was equal to 9 (n = 9), and the mean and the standard deviations were calculated in Excel.

## Data Availability

All information required to reanalyze the data in this report is presented in the Supporting Information or from the lead contact upon request.

## References

[1] Wartell B., Boufadel M., Rodriguez-Freire L. (2021). An effort to understand and improve the anaerobic biodegradation of petroleum hydrocarbons: a literature review. Int. Biodeterior. Biodegrad..

[2] Laczi K., Erdeiné Kis Á., Szilágyi Á., Bounedjoum N., Bodor A., Vincze G.E., Kovács T., Rákhely G., Perei K. (2020). New frontiers of anaerobic hydrocarbon biodegradation in the multi-omics era. Front. Microbiol..

[3] Zhang Y., Zhai X., Guan F., Dong X., Sun J., Zhang R., Duan J., Zhang B., Hou B. (2022). Microbiologically influenced corrosion of steel in coastal surface seawater contaminated by crude oil. Npj Mater. Degrad..

[4] Widdel F., Musat F., Rojo F. (2019). Aerobic Utilization of Hydrocarbons, Oils, and Lipids.

[5] Backman L.R.F., Funk M.A., Dawson C.D., Drennan C.L. (2017). New tricks for the glycyl radical enzyme family. Crit. Rev. Biochem. Mol. Biol..

[6] Funk M.A., Judd E.T., Marsh E.N.G., Elliott S.J., Drennan C.L. (2014). Structures of benzylsuccinate synthase elucidate roles of accessory subunits in glycyl radical enzyme activation and activity. Proc. Natl. Acad. Sci. USA.

[7] Funk M.A., Marsh E.N.G., Drennan C.L. (2015). Substrate-bound structures of benzylsuccinate synthase reveal how toluene is activated in anaerobic hydrocarbon degradation. J. Biol. Chem..

[8] Himo F. (2002). Catalytic mechanism of benzylsuccinate synthase, a theoretical study. J. Phys. Chem. B.

[9] Qiao C., Marsh E.N.G. (2005). Mechanism of benzylsuccinate synthase: stereochemistry of toluene addition to fumarate and maleate. J. Am. Chem. Soc..

[10] Li L., Marsh E.N.G. (2006). Mechanism of benzylsuccinate synthase probed by substrate and isotope exchange. J. Am. Chem. Soc..

[11] Peng Y., Veneziano S.E., Gillispie G.D., Broderick J.B. (2010). Pyruvate formate-lyase, evidence for an open conformation favored in the presence of its activating enzyme. J. Biol. Chem..

[12] Shisler K.A., Broderick J.B. (2014). Glycyl radical activating enzymes: structure, mechanism, and substrate interactions. Arch. Biochem. Biophys..

[13] Knappe J., Neugebauer F.A., Blaschkowski H.P., Gänzler M. (1984). Post-translational activation introduces a free radical into pyruvate formate-lyase. Proc. Natl. Acad. Sci. USA.

[14] Vey J.L., Yang J., Li M., Broderick W.E., Broderick J.B., Drennan C.L. (2008). Structural basis for glycyl radical formation by pyruvate formate-lyase activating enzyme. Proc. Natl. Acad. Sci. USA.

[15] Krebs C., Broderick W.E., Henshaw T.F., Broderick J.B., Huynh B.H. (2002). Coordination of adenosylmethionine to a unique iron site of the [4Fe-4S] of pyruvate formate-lyase activating enzyme: a Mössbauer spectroscopic study. J. Am. Chem. Soc..

[16] Walsby C.J., Hong W., Broderick W.E., Cheek J., Ortillo D., Broderick J.B., Hoffman B.M. (2002). Electron-nuclear double resonance spectroscopic evidence that *S* -adenosylmethionine binds in contact with the catalytically active [4Fe−4S] ^+^ cluster of pyruvate formate-lyase activating enzyme. J. Am. Chem. Soc..

[17] Byer A.S., Yang H., McDaniel E.C., Kathiresan V., Impano S., Pagnier A., Watts H., Denler C., Vagstad A.L., Piel J. (2018). Paradigm shift for radical *S*-Adenosyl-L-methionine reactions: the organometallic intermediate Ω is central to catalysis. J. Am. Chem. Soc..

[18] Becker A., Fritz-Wolf K., Kabsch W., Knappe J., Schultz S., Volker Wagner A.F. (1999). Structure and mechanism of the glycyl radical enzyme pyruvate formate-lyase. Nat. Struct. Biol..

[19] Leppänen V.M., Merckel M.C., Ollis D.L., Wong K.K., Kozarich J.W., Goldman A. (1999). Pyruvate formate lyase is structurally homologous to type I ribonucleotide reductase. Structure.

[20] Selmer T., Andrei P.I. (2001). p-Hydroxyphenylacetate decarboxylase from Clostridium difficile. A novel glycyl radical enzyme catalysing the formation of *p*-cresol. Eur. J. Biochem..

[21] Leuthner B., Leutwein C., Schulz H., Hörth P., Haehnel W., Schiltz E., Schägger H., Heider J. (1998). Biochemical and genetic characterization of benzylsuccinate synthase from *Thauera aromatica*: a new glycyl radical enzyme catalysing the first step in anaerobic toluene metabolism. Mol. Microbiol..

[22] Martins B.M., Blaser M., Feliks M., Ullmann G.M., Buckel W., Selmer T. (2011). Structural basis for a Kolbe-type decarboxylation catalyzed by a glycyl radical enzyme. J. Am. Chem. Soc..

[23] Li L., Patterson D.P., Fox C.C., Lin B., Coschigano P.W., Marsh E.N.G. (2009). Subunit structure of benzylsuccinate synthase. Biochemistry.

[24] Hilberg M., Pierik A.J., Bill E., Friedrich T., Lippert M.-L., Heider J. (2012). Identification of FeS clusters in the glycyl-radical enzyme benzylsuccinate synthase via EPR and Mössbauer spectroscopy. J. Biol. Inorg. Chem..

[25] Coschigano P.W. (2002). Construction and characterization of insertion/deletion mutations of the tutF, tutD, and tutG genes of *Thauera aromatica* strain T1. FEMS Microbiol. Lett..

[26] Salii I., Szaleniec M., Zein A.A., Seyhan D., Sekuła A., Schühle K., Kaplieva-Dudek I., Linne U., Meckenstock R.U., Heider J. (2021). Determinants for substrate recognition in the glycyl radical enzyme benzylsuccinate synthase revealed by targeted mutagenesis. ACS Catal..

[27] Biegert T., Fuchs G., Heider J. (1996). Evidence that anaerobic oxidation of toluene in the denitrifying bacterium *Thauera aromatica* is initiated by formation of benzylsuccinate from toluene and fumarate. Eur. J. Biochem..

[28] Beller H.R., Spormann A.M. (1998). Analysis of the novel benzylsuccinate synthase reaction for anaerobic toluene activation based on structural studies of the product. J. Bacteriol..

[29] Frey M., Rothe M., Wagner A.F., Knappe J. (1994). Adenosylmethionine-dependent synthesis of the glycyl radical in pyruvate formate-lyase by abstraction of the glycine C-2 pro-S hydrogen atom. Studies of [2H]glycine-substituted enzyme and peptides homologous to the glycine 734 site. J. Biol. Chem..

[30] Harms G., Rabus R., Widdel F. (1999). Anaerobic oxidation of the aromatic plant hydrocarbon *p*-cymene by newly isolated denitrifying bacteria. Arch. Microbiol..

[31] Strijkstra A., Trautwein K., Jarling R., Wöhlbrand L., Dörries M., Reinhardt R., Drozdowska M., Golding B.T., Wilkes H., Rabus R. (2014). Anaerobic activation of *p-*cymene in denitrifying betaproteobacteria: methyl group hydroxylation versus addition to fumarate. Appl. Environ. Microbiol..

[32] Selvaraj B., Pierik A.J., Bill E., Martins B.M. (2013). 4-Hydroxyphenylacetate decarboxylase activating enzyme catalyses a classical *S*-adenosylmethionine reductive cleavage reaction. J. Biol. Inorg. Chem..

[33] Selvaraj B., Pierik A.J., Bill E., Martins B.M. (2014). The ferredoxin-like domain of the activating enzyme is required for generating a lasting glycyl radical in 4-hydroxyphenylacetate decarboxylase. J. Biol. Inorg. Chem..

[34] Wang Y., Nguyen N., Lee S.H., Wang Q., May J.A., Gonzalez R., Cirino P.C. (2022). Engineering *Escherichia coli* for anaerobic alkane activation: biosynthesis of (1-methylalkyl)succinates. Biotechnol. Bioeng..

[35] McCarthy E.L., Booker S.J. (2018). Biochemical approaches for understanding iron–sulfur cluster regeneration in *Escherichia coli* lipoyl synthase during catalysis.. Methods Enzymol..

[36] Backman L.R., Huang Y.Y., Andorfer M.C., Gold B., Raines R.T., Balskus E.P., Drennan C.L. (2020). Molecular basis for catabolism of the abundant metabolite trans-4-hydroxy-L-proline by a microbial glycyl radical enzyme. Elife.

