# Supplementary material for: Development of an in vitro method for activation of X-succinate synthases for fumarate hydroalkylation
Source: iScience. 2023 May 19;26(6):106902. doi: 10.1016/j.isci.2023.106902 (PMC10239695; doi:10.1016/j.isci.2023.106902)
Supplement: Document S1. Figures S1–S7 and Tables S1–S8 [file mmc1.pdf]

iScience, Volume *26*

Supplemental information

Development of an *in vitro* method for activation of X-succinate synthases for fumarate hydroalkylation

Mary C. Andorfer, Devin T. King-Roberts, Christa N. Imrich, Balyn G. Brotheridge, and Catherine L. Drennan

SI Tables

Table S1: Percent identity matrix, calculated using Clustal2.1 (related to STAR Methods).[1] Color coded according to % identity (dark red, 100%; light red, 80–99%; orange, 60–79%; yellow, 40–59%).

**Entry 5** (UPI0012D2EB72)	100.00	54.06	54.72	54.38	55.62	58.26	58.57
**3** (A0A096ZNX5)	54.02	100.00	65.20	60.62	58.89	66.04	59.77
**2** (UPI000BBC9D3F)	54.72	65.20	100.00	62.58	63.21	66.14	65.20
**4** (A0A1H5T9V3)	54.38	60.62	62.58	100.00	80.36	69.18	60.12
**6** (Q5P6A4)	55.62	58.89	63.21	80.36	100.00	70.39	56.71
**1** (I6LHT6)	58.26	66.04	66.14	69.18	70.39	100.00	76.81
BSS-AE$_{Ta}$ (O68393)	58.57	59.77	65.20	60.12	56.71	76.81	100.00

Table S2: BSS-AE$_{Ta}$ homologs screened for solubility in *E. coli* in this study (related to STAR Methods).

**Entry**	**Organism**	**Gene identifier**	**Gene name**	**Forward primer (5′->3′)**	**Reverse primer (5′->3′)**
**1**	*Azoarcus sp. CIB*	I6LHT6 (UniProt ID)	bssD	CGTATTCCTC TGGTCACAG	CATGGTATATCT CCTTCTTAAAG
**2**	*Magnetospirillum sp. 15-1*	UPI000BBC9D3F (UniParc ID)	bssD	AAGATCCCCT TGATTACAG	CATGGTATATCT CCTTCTTAAAG
**3**	*Thauera sp. pCyN*	A0A096ZNX5 (UniProt ID)	ibsD	ATGCACATCG ATGGCACC	CATGGTATATCTCCT TCTTAAAGTTAAAC
**4**	*Thauera chlorobenzoica*	A0A1H5T9V3 (UniProt ID)	Tchl_1407	AAAATCCCCT TGGTAACTG	CATGGTATATCT CCTTCTTAAAG
**5**	*Desulfosarcina ovata*	UPI0012D2EB72 (UniParc ID)	N/A	AAGATTCCA CTGATCACG	CATGGTATATCTC CTTCTTAAAG
**6**	*Aromatoleum aromaticum strain EbN1*	Q5P6A4 (UniProt ID)	bssD	CTTAAGTACA CGACTAGTAC	CATGGTATATCTC CTTCTTAAAG

Table S3: Averages and standard deviations plotted in Figure 4B comparing end point AdoMet cleavage assays by IbsAE.

Condition	Average [dAdo] (μM)	Stdev
No AE, No IBSS	-0.047	0.051
As purified IbsAE, No IBSS	1.122	0.036
Reconstituted IbsAE, No IBSS	1.373	0.094
No AE, IBSSαγ	0.170	0.100
As purified IbsAE, IBSSαγ	47.620	2.346
Reconstituted IbsAE, IBSSαγ	46.702	1.691
No AE, IBSSαβγ	0.100	0.160
As purified IbsAE, IBSSαβγ	7.164	0.495
Reconstituted IbsAE, IBSSαβγ	5.444	0.266

Table S4: Averages and standard deviations plotted in Figure 4C comparing AdoMet cleavage time course assays by IbsAE.

Time (min)	as purified IbsAE, IBSSαγ Average [dAdo] (μM)	as purified IbsAE, IBSSαβγ Average [dAdo] (μM)
1.5	0.99 ± 0.15	0.18 ± 0.55
15	2.44 ± 0.16	0.34 ± 0.04
30	6.85 ± 0.19	0.75 ± 0.05
45	11.96 ± 0.22	1.32 ± 0.04
60	15.82 ± 0.07	2.06 ± 0.05
75	20.41 ± 0.72	2.92 ± 0.17
90	24.64 ± 1.13	3.74 ± 0.17
105	28.31 ± 1.27	4.51 ± 0.17
120	32.39 ± 1.16	5.64 ± 0.32

Table S5: EPR quantification of glycyl radical installation within IBSS with IbsAE plotted in Figure 5B and Figure S6. Double integrals of EPR spectra were calculated using Xenon software and compared to double integrals of known concentrations of Fremy's salt standards to calculate concentration of radical in μM.

Time (min)	IbsAE as purified μM radical	IbsAE reconstituted μM radical	IbsAE as purified + IBSSβ μM radical
21	4.46	0.79	0.35
41	9.15	0.75	0.28
61	11.61	1.11	0.28
82	14.84	1.46	0.01
101	16.33	1.45	0.39
121	16.36	2.73	0.51

Table S6: EPR quantification of glycyl radical installation within IBSS with IbsAE plotted in Figure 5C. Double integrals of EPR spectra were calculated using Xenon software and compared to double integrals of known concentrations of Fremy's salt standards to calculate concentration of radical in μM.

**Time (h)**	**μM radical**
0.5	11.64
1	17.21
2	22.98
3	23.03
4	20.23
5	22.89
6	21.19

Table S7: EPR quantification of glycyl radical installation within BSS with IbsAE plotted in Figure 6B. Double integrals of EPR spectra were calculated using Xenon software and compared to double integrals of known concentrations of Fremy's salt standards to calculate concentration of radical in μM.

**Time (h)**	**μM radical, BSSαγ**	**μM radical, BSSαβγ**
0.333	3.93	0.61
0.667	7.10	2.25
1	9.66	2.45
1.333	11.78	2.33
1.667	16.09	3.40
2	16.66	3.31
3	22.11	-
4	21.05	4.33

Table S8: Benzylsuccinate formation under different conditions (related to Figure 7). The LCMS assay yields are plotted in Fig 7 and were calculated by integrating the EIC peak for benzylsuccinate and comparing to a standard curve made from authentic standard. The integration values for the EIC for fumarate (one of the starting materials) are also shown. When BSSαβγ is activated and added to the reactions, all fumarate is consumed.

BSSαβγ		BSSαγ (no BSSβ)		No BSS	
% Assay Yield	fumarate (EIC)	% Assay Yield	fumarate (EIC)	% Assay Yield	fumarate (EIC)
92.44	0	0.60	147105	0.06	182475
93.56	0	0.68	138095	0	176621
88.78	0	0.61	138117	0	173527
92.41	0	0.69	135287	0	168892
92.87	0	0.66	137080	0.01	161573
92.47	0	0.65	132851	0	157939
97.42	0	0.69	140342	0	163441
90.33	0	0.68	135425	0	159362
90.24	0	0.63	118804	0	165476

SI Figures

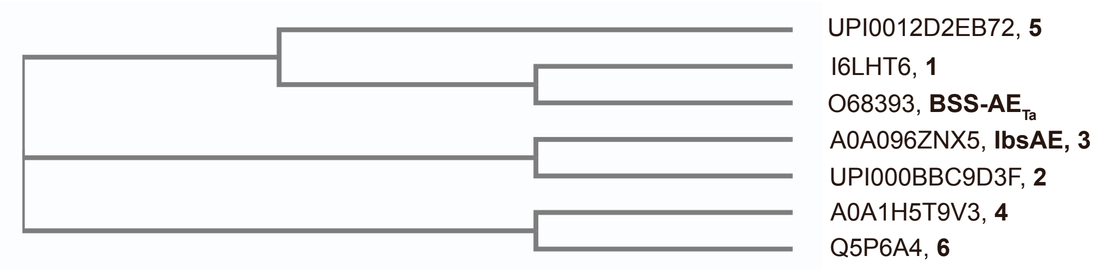

Figure S1: Phylogenetic tree of XSS-AE homologs, calculated using Clustal2.1 (related to STAR Methods).[1]

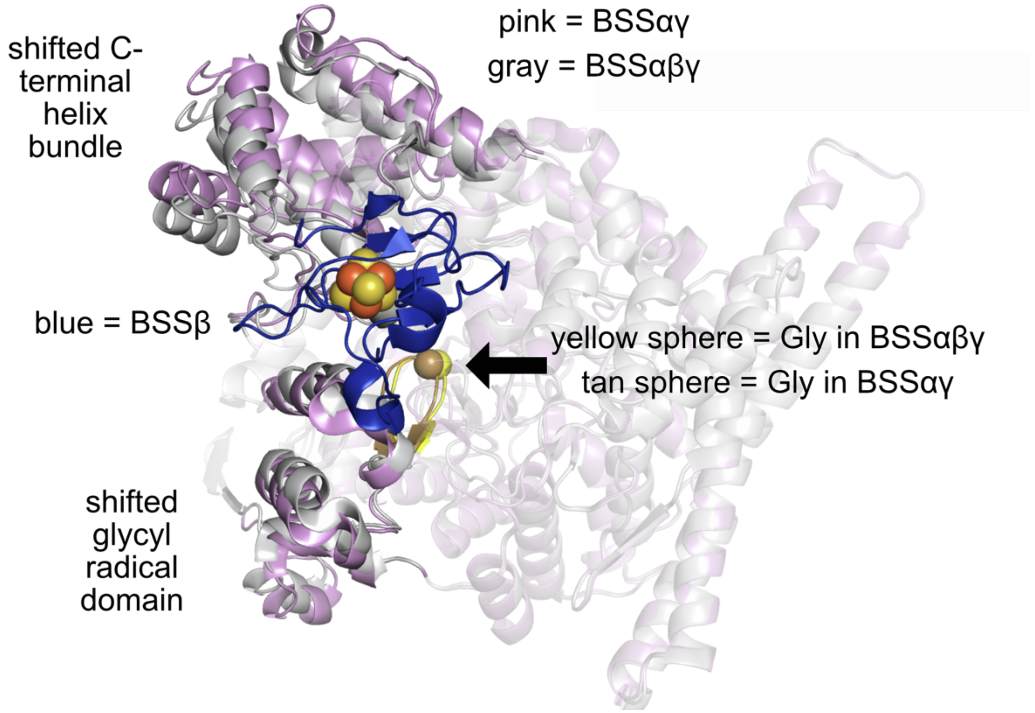

Figure S2: Previous crystal structures of BSSαγ (pink, PDB ID 4PKC[2]) and BSSαβγ (grey, PDB ID 4PKF[2]) provided a proposal for the role of the BSSβ subunit (blue, PDB ID 4PKF[2]) (related to Figure 1). When the two structures are aligned and compared, a more open conformation is observed when BSSβ is not present. In the more open conformation, the C-terminal helix bundle and glycyl radical domain expand in a clam-shell like motion.

Ferredoxin domain | Active site Cys | Ferredoxin domain Cys

```
BSS-AE-Ta       MVVVDRPPPPDLGGGVIVRNECSHSEPIDTGRMTFPTCDSGADVKIPLVTEIQRFSLQDG     60
IbsAE           MM--------HIDGTVGWQDALSSSEDDEGQCCEFDTHTL---GRTIQVTEIQRFSLQDG     49
                *:        .:.* *  ::    * **  :   * *       :   ************

BSS-AE-Ta       PGFRTTVFLKGCPLRCPWCHNPETQKVGKEYYYNRDRCVSCGRCATVCPTGASQLLDGPG     120
IbsAE           PGIRTTIFLKGCPLRCPWCHNPETQDLHQELYYYRSRCTGCGRCAAVCPSGASTLVKGAD     109
                **:***:******************.: :* ** *.**..*****:***:*** *:.* .

BSS-AE-Ta       ASQVLKLDRSKCINCMRCVAVCLTGSRDSVGMEMTLDEILREVLSDEPFYRNSGGGVTIS     180
IbsAE           GRPTLNLDRSKCEKCMRCVSACLSSARAIAGQSLSIDDILREALSDRLFYDNSGGGVTLS     169
                .  .*:****** :*****:.**:.:*  .* .:::*:****.***. ** *******:*

BSS-AE-Ta       GGDPLFHPAFTLELARKIKERGVHVAIETSCFPKKWATIQPLLKLVDLFIVDLKSLNRKK     240
IbsAE           GGDPLRQPEASLELARRIKAEGVHVAMETSCFPKRWQTIEPLLEVIDLFIVDLKTLDAEK     229
                ***** :*  :*****:** .*****:*******:* **:***::********:*:  :*

BSS-AE-Ta       HEETVGWPLQPILDNIEHLIQAKANIRIHIPVIPGFNDSPMDFEDYIAYLGRHAAQLDGV     300
IbsAE           HEKVIRWPLAPILRNLDRLIESGANVRIHIPIVPGFNDSEKDFADFVAFLSRYLGRLNGI     289
                **:.: *** *** *:::**:: **:*****::******  ** *::*:*.*: .:*:*:

BSS-AE-Ta       DILNYHVYGEGKYRSLGRENEYQYFGVEENPPEKVVPLAKGLKLAGITSVTIGGLVGITA     360
IbsAE           DILNYHSYAVSKYDALGRGEAYAYREVEENPPEKVLPLAQSLKQAGFESVTIGGLVGITT     349
                ****** *. .** :*** : * *  *********:***:.** **: ***********:

BSS-AE-Ta       DRHKSSRDAGTGCIA  375
IbsAE           AGSE-----------  353
                   :
```

Figure S3: Sequence alignment of BSS-AE from *Thauera aromatica* (Uniprot ID: O68393) and IbsAE from *Thauera* sp. pCyN (Uniprot ID: A0A096ZNX5) using CLUSTAL O (1.2.4) multiple sequence alignment (related to STAR Methods).[1] The ferredoxin domain, which is not observed in the only structurally characterized GRE-AE (PFL-AE, PDB ID: 3CB8[3]), is shown in grey. Cysteine residues predicted to ligate the active site [4Fe–4S] cluster are highlighted in yellow. Cysteine residues predicted to ligate [4Fe–4S] clusters in the ferredoxin domain are highlighted in orange.

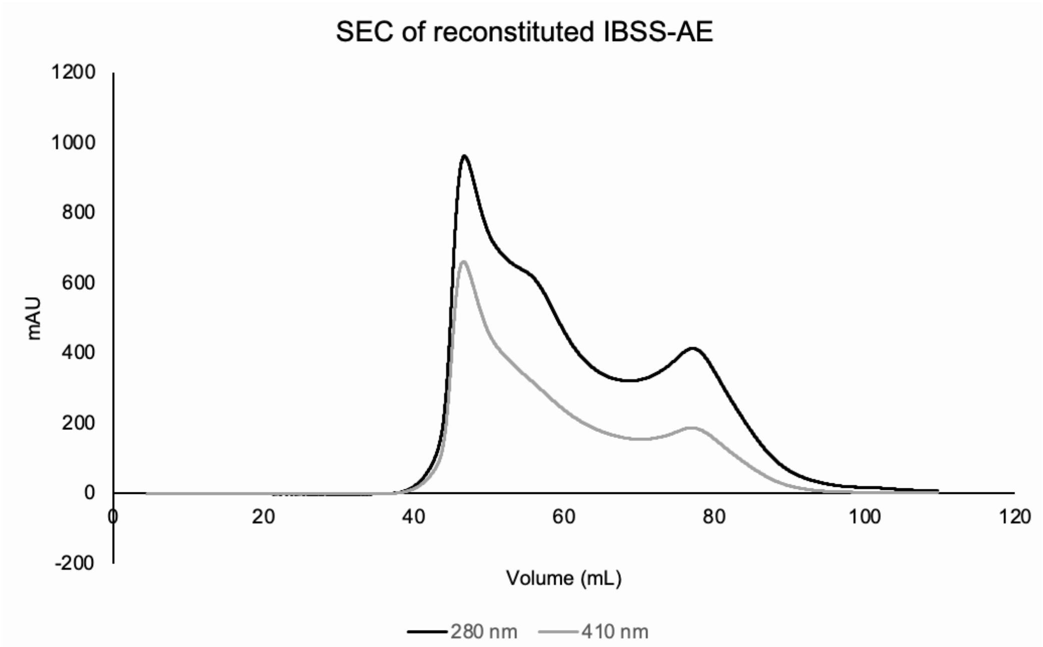

Figure S4: Trace from size exclusion chromatography (SEC) after reconstitution of IbsAE (related to STAR Methods). The black line is absorbance units at 280 nm and the grey line is absorbance units at 410 nm. The large peak at ~45-50 mL is aggregated IbsAE and was discarded. The peak around ~78 mL is IbsAE monomer and was collected for assays.

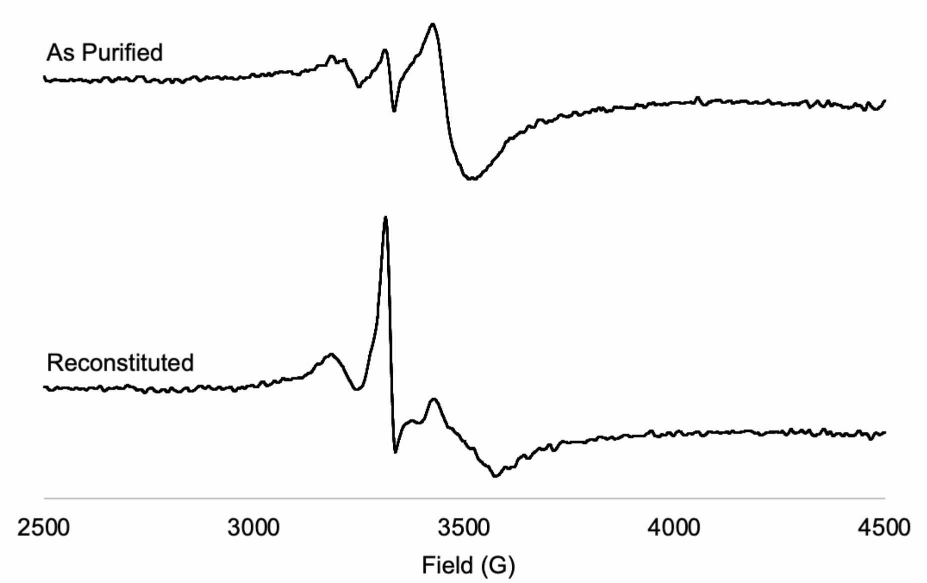

Figure S5: X-band EPR spectra of photoreduced IbsAE in the presence of 5-deazariboflavin before and after reconstitution (related to STAR Methods). A mix of signals was observed, corresponding to [4Fe–4S] cluster and [3Fe–4S] cluster. More [3Fe–4S] cluster was observed in the reconstituted IbsAE relative to the as purified IbsAE. Conditions of measurement: T = 10 K; microwave power = 50 μW; microwave frequency = 9.37 GHz; modulation amplitude = 10 G; [IbsAE] = 60 μM.

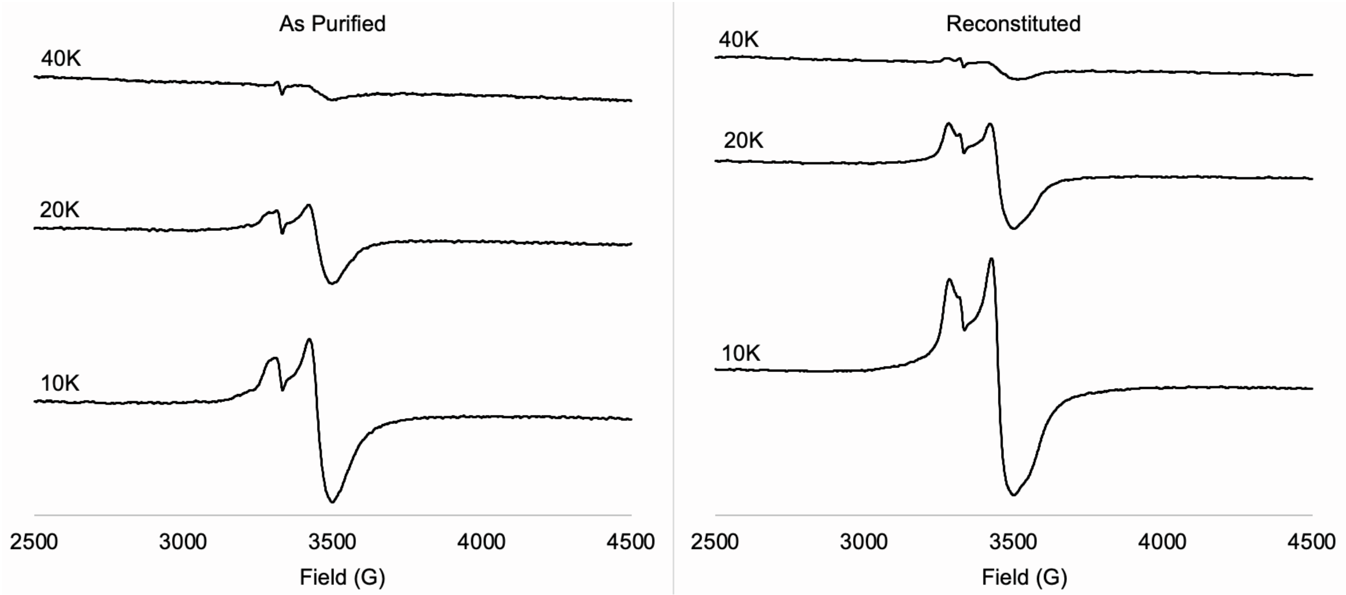

Figure S6: Temperature studies of IbsAE before (as purified) and after (reconstituted) reconstitution of FeS clusters (related to STAR Methods). IbsAE was incubated with dithionite (1 mM) for 1 hour prior to freezing. X-band EPR spectra of IbsAE are shown at 10, 20, and 40 K. The primary signal observed in both samples is $[4Fe–4S]^{+}$, as indicated by the disappearance of signal at higher temperatures. Conditions of measurement: T = 10–40 K; microwave power = 50 μW; microwave frequency = 9.37 GHz; modulation amplitude = 10 G; [IbsAE] = 60 μM.

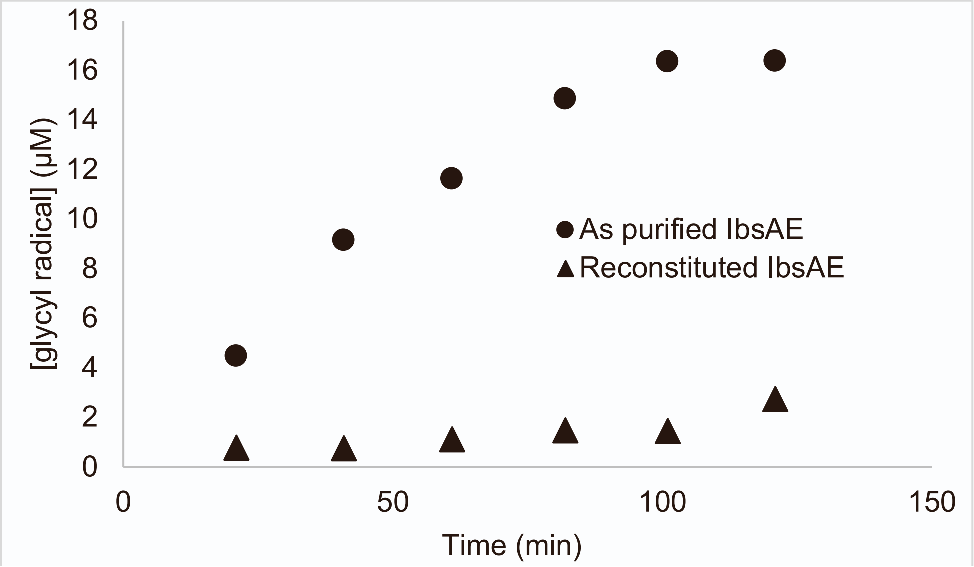

Figure S7: EPR quantification of glycyl radical installation within IBSSαγ with IbsAE before (as purified) and after (reconstituted) reconstitution (related to Figure 5). Conditions of measurement: T = 80 K; microwave power = 1.26 μW; microwave frequency = 9.37 GHz; modulation amplitude = 3 G; [IBSSαγ] = 50 μM; [IbsAE] = 50 μM.

1. Sievers, F., Wilm, A., Dineen, D., Gibson, T.J., Karplus, K., Li, W., Lopez, R., McWilliam, H., Remmert, M., Söding, J., et al. (2011). Fast, scalable generation of high-quality protein multiple sequence alignments using Clustal Omega. Mol. Syst. Biol. *7*, 539. 10.1038/msb.2011.75.

2. Funk, M.A., Judd, E.T., Marsh, E.N.G., Elliott, S.J., and Drennan, C.L. (2014). Structures of benzylsuccinate synthase elucidate roles of accessory subunits in glycyl radical enzyme activation and activity. Proc. Natl. Acad. Sci. *111*, 10161–10166. 10.1073/pnas.1405983111.

3. Vey, J.L., Yang, J., Li, M., Broderick, W.E., Broderick, J.B., and Drennan, C.L. (2008). Structural basis for glycyl radical formation by pyruvate formate-lyase activating enzyme. Proc. Natl. Acad. Sci. *105*, 16137–16141. 10.1073/pnas.0806640105.
